# Retinal Pigment Epithelium and Neural Retinal Progenitors Interact via Semaphorin 6D to Facilitate Optic Cup Morphogenesis

**DOI:** 10.1523/ENEURO.0053-21.2021

**Published:** 2021-05-06

**Authors:** Paula Bernice Cechmanek, Carrie Lynn Hehr, Sarah McFarlane

**Affiliations:** 1 Graduate Program in Neuroscience, University of Calgary, Calgary, Alberta T2N 1N4, Canada; 2 Department of Cell Biology and Anatomy, Hotchkiss Brain Institute, Alberta Children’s Hospital Research Institute, University of Calgary, Calgary, Alberta T2N 1N4, Canada

**Keywords:** *Danio rerio*, eye, Plexina1, repulsion, RPE, zebrafish

## Abstract

Cell movement propels embryonic tissues to acquire shapes required for mature function. The movements are driven both by acto-myosin signaling and by cells interacting with the extracellular matrix (ECM). Unknown is whether cell-cell interactions within a tissue are also required, and the molecular mechanisms by which such communication might occur. Here, we use the developing visual system of zebrafish as a model to understand the role cell-cell communication plays in tissue morphogenesis in the embryonic nervous system. We identify that cell-cell-mediated contact between two distinct cell populations, progenitors of the neural retina and retinal pigment epithelium (RPE), facilitates epithelial flow to produce the mature cupped retina. We identify for the first time the need in eye morphogenesis for distinct populations of progenitors to interact, and suggest a novel role for a member of a key developmental signaling family, the transmembrane Semaphorin6d, as mediating communication between distinct cell types to control tissue morphogenesis.

## Significance Statement

We identify that cell-cell communication between neural progenitors in the embryonic eye and their non-neural, retinal pigmented epithelial cell neighbors promotes the cell movements which are required to form a cupped retina that mediates vision. We also reveal the repulsive guidance molecule Semaphorin6d as the molecular mechanism that allows these two distinct cell populations to work together to orchestrate optic cup morphogenesis.

## Introduction

During development individual tissues undergo morphogenesis driven by cell movements (for review, see Cavodeassi, 2018; Moreno-Marmol et al., 2018). Morphogenesis ensures that the tissue takes on the necessary shape with specific cell types in their proper locations so that the organ can carry out its appropriate function. This is true of the eyes, where during embryogenesis eye vesicles emerge from the forebrain and undergo extensive morphogenesis to produce a cupped retina (optic cup). The outer leaflet of the optic cup comprises the neurons of the retina, while the inner leaflet consists of the retinal pigment epithelium (RPE; Heermann et al., 2015) that supports the photoreceptors (for review, see Bharti et al., 2011). The cell interactions and molecular mechanisms that drive optic cup morphogenesis, however, are poorly understood.

With the zebrafish model, the speed of development, the transparency of the embryos, and fluorescent cell specific transgenic lines, have allowed for visualization of eye progenitor movements in the live embryo over the period of eye morphogenesis (Picker et al., 2009; Kwan et al., 2012; Ivanovitch et al., 2013; Heermann et al., 2015; Cechmanek and McFarlane, 2017). These studies revealed the cellular movements that drive both vesicle evagination and elongation (Ivanovitch et al., 2013), and the second phase of eye morphogenesis, where neural retina progenitors of the inner leaflet of the eye vesicle move around the distal rim of the forming optic cup into the outer leaflet to become the temporal neural retina (Picker et al., 2009; Kwan et al., 2012; Heermann et al., 2015; Sidhaye and Norden, 2017). As a consequence, the RPE stretches around the back of the neural retina (Kwan et al., 2012; Cechmanek and McFarlane, 2017). The cupping of the neural retina depends on the apical constriction of retinal progenitors and Integrin signaling (Nicolás-Pérez et al., 2016; Sidhaye and Norden, 2017), while movement around the rim requires interactions of neural progenitors with Laminin on the basal surface of the eye neuroepithelium (Sidhaye and Norden, 2017). Unclear is whether the movements of the cells of the inner eye vesicle leaflet depend on contact between RPE and neural retina progenitors, and if so, what molecules mediate the interactions.

Semaphorins (Semas) are a large family of secreted, membrane-associated and membrane-tethered ligands, of which classes 3–7 are present in vertebrates (for review, see Raper, 2000). Semas play repellent roles, and direct cells and/or their neurites away from the Sema source, but can also control proliferation, neurite outgrowth, and act as attractants (for review, see Battistini and Tamagnone, 2016; Alto and Terman, 2018). In the developing visual system, different members of the secreted Sema3 family control the growth of retinal ganglion cell axons and dendrites (Sakai and Halloran, 2006; Atkinson-Leadbeater et al., 2010; Kita et al., 2013), while transmembrane Sema6a controls eye progenitor movements in the early eye vesicle (Ebert et al., 2014). Here, we use the zebrafish model to explore a novel role for transmembrane Sema6s as regulators of tissue morphogenesis in the developing nervous system. We find the transmembrane ligand, Sema6d, and its known receptor, Plexina1a (Plxna1a), are expressed in a complementary pattern in the inner leaflet of the eye vesicle before the onset of optic cup morphogenesis; with transcripts for *sema6d* and *plxna1a* in neural retinal and RPE progenitors, respectively. Loss-of-function of *sema6d* by CRISPR/Cas9-mediated mutagenesis causes defects in temporal but not nasal optic cup morphogenesis, a phenotype that is reproduced by transient knock down of Plxna1a, and by blockade of a known Sema6d signaling effector, c-Abl (Kang et al., 2018). With loss of Sema6d signaling, morphogenesis of the temporal eye initiates, but then stalls: neural progenitors of the inner leaflet fail to move properly into the retina, and RPE progenitors fail to come to abut the lens. These data reveal for the first time the importance of cell-cell interactions in optic cup morphogenesis, and identify transmembrane Sema6s as functioning in neural progenitors to facilitate tissue morphogenesis during nervous system development.

## Materials and Methods

### Animals

Tupfel Longfin (TL; ZIRC) embryos were raised at 28°C in E3 medium supplemented with 0.25 mg/l methylene blue as described previously (Westerfield, 2000). Embryos were staged by number of somites up to the 18 somite stage (ss), and by hours postfertilization (hpf) thereafter (Kimmel et al., 1995). The *Tg(rx3:GFP)* fish were gifted from the Wittbrodt lab (Rembold et al., 2006) and the *Tg(tfec:EGFP)* line was regenerated using a Tol2 construct provided by Brian Link (Miesfeld et al., 2015). *Tg(vsx1:GFP)* fish were generated by the Higashijima lab (Kimura et al., 2008). Zero hpf is designated as the time dividers were removed for breeding, shortly after lights on in the fish facility. The University of Calgary Animal Care Committee approved the animal protocols.

### *sema6d* mutants

*sema6d* mutant lines were created with CRISPR/Cas9 mutagenesis techniques according to (Gagnon et al., 2014) with the use of online gene editing tools [CHOPCHOP: www.chopchop.cbu.uib.no (Montague et al., 2014); ViennaRNA fold server: http://rna.tbi.univie.ac.at/, CRISPR Design: www.crispr.mit.edu]. A total of 12.5 pg of sgRNA (exon-6 sgRNA: 5′- CATTTAGGTGACACTATAGAGTTTACTCACTCTGTAGTTGGTTTTAGAGCTAGAAATAGCAAG-3′, and exon-10 sgRNA: 5′- CATTTAGGTGACACTATAGAGGTGAACACTCCGACTACAGGTTTTAGAGCTAGAAATAGCAAG-3′) alongside 150- to 300-pg mRNA encoding Cas9 (Addgene) or 600-pg Cas9 protein (PNA Bio) was injected into one cell-stage embryos that were then grown to adulthood and outcrossed to screen for germ-line mutations via PCR product size. Candidate mutants were cloned and sequenced around the sgRNA target site and two alleles were identified; the exon6 mutant line *sema6d^ca302/ca302^* and the exon10 mutant line *sema6d^ca303/ca303^*.

### Inhibition experiments

For morpholino oligonucleotide (MO)-mediated knock down of Plxna1, 1 nl of 125 μm *plxna1a* antisense MO targeting the exon2-intron2 splice site (5′-TGAGGCGAGATTTACCTTGGATATT-3′; Gene Tools) or control water solution was injected at the one-cell stage into wild-type TL embryos and splice knock-down was verified using PCR primers (forward 5′-ACTGCCCTGCGTTTTAACAG-3′ and reverse 5′-AGCGAGCACTTCTTCTCCTG-3′). For CRISPRi (Gilbert et al., 2013; Larson et al., 2013) against *plxna1a*, 1 nl of solution containing 12.5 ng/μL of the exon5-targeting sgRNA 5′- CATTTAGGTGACACTATAGACTCTCACGCAAACCTCTACTGTTTTAGAGCTAGAAATAGCAAG-3′ and 150 ng/μl of mRNA encoding deadCas9 (Addgene) were injected into one cell-stage embryos (or deadCas9 mRNA only for control injections) and transcript knock-down was verified with 24-hpf mRNA using primers forward 5′-ATCTCGGCGACAGAGACTGC-3′ and reverse 5′-TGGCATCCGGGGAGACATTC-3′. β-Actin control primers used were forward 5′-GCAGAAGGAGATCACATCCCTGGC-3′ and reverse 5′-CATTGCCGTCACCTTCACCGTTC-3′ (Casadei et al., 2011).

*sema6dex6+/+* and *sema6d^ca302^*embryos at the 16 ss were incubated either in control solution or in 100 μm dasatinib (Selleckchem), a pharmacological inhibitor known to block c-Abl activity (Lombardo et al., 2004). At 24 hpf (7 h later), embryos were fixed in 4% paraformaldehyde (PFA) overnight, dehydrated, and stored in methanol at −20°C, before processing for whole-mount *in situ* hybridization (ISH) with an antisense probe for *vax2*.

### Protein and gene expression

Wild-type, mutant, and treated embryos that showed significant developmental delay (>1 ss) were excluded before fixation and analysis. Digoxygenin (DIG)-labeled antisense riboprobes were synthesized as described previously from plasmid templates (Table 1; Thisse and Thisse, 2014) and colorimetric RNA ISH was performed by using whole embryos as outlined previously (Tessmar-Raible et al., 2005; Thisse and Thisse, 2014), with the following modifications: (1) embryos analyzed at 24 hpf were treated with 0.003% (w/v) 1-phenyl-2-thiourea (Sigma) to prevent pigment biosynthesis; (2) embryos were permeabilized with 2% hydrogen peroxide (Sigma) in methanol for 20 min and Proteinase-K was not used; (3) embryos were hybridized over night at 70°C with 1/100 dilution of antisense riboprobe plus 5% dextran sulfate (Alfa Aeser) in hybridization buffer, and 4) 2.25 μl/ml nitro-blue tetrazolium chloride (NBT; Roche) and 3.5 μl/ml 5-bromo-4-chloro-3′-indolyphosphate p-toluidine salt (BCIP; Roche) were heated at 50°C for 5 min before using for the staining reaction. For immunostaining, 12-μm transverse cryostat (Microm) sections were made of 4% PFA-fixed 16- to 18-ss embryos embedded in optimal cutting temperature mounting media (Sakura), and collected on glass slides. Sections were processed for immunostaining with the following antibodies; a rabbit polyclonal against the extracellular domain of human PLXNA1 (Alomone, catalog #APR-081, lot #Apr081AN0125), followed by an Alexa Fluor 488 secondary antibody (Invitrogen), and c-Abl (phospho-Tyr245; Aviva Systems Biology) at 1:50. c-Abl immunolabeling involved a two-step secondary; unconjugated mouse anti-rabbit at 1:1000 (Jackson) and Alexa Fluor 555 anti-mouse at 1:1000.

**Table 1:** Antisense riboprobes used for RNA ISH

Probe	Primers (5′−3′)	Linearize/transcribe
*bhlhe40*	F-TTGCAAATCGGCGAACAGGG R-GGAAACGTGCACGCAGTCG	EcoRV/SP6
*foxd1*	F-AGGCAACTACTGGACGCTAGACCCTG R-GAAACAGACCGTGTAAAAATATCACACTCCGAG	EcoRV/SP6
*foxg1a*	F-GCAGGAAGAAAAACGGGACGC R-GATGGGTGAGGGACATGGGG	EcoRV/SP6
*plxna1a*	F-ACGGGTCAGTTATCGCCCTG R-CGCCGACAGGATCTCGTCTT	EcoRV/SP6
*plxna1b*	F-CTCAGCCGGAAAACACATGG R-GAACTTCACCTCCGGGTTTCTG	EcoRV/SP6
*sema6d*	F-GCAGTAGCAGTCAACGTTCTG R-GGGGTCAGTAGTTGTGTGTCGT	BamH1/T7
*tbx5*	F-GGAGCTGCATCGCATGTCAC R-TGTCCAGTGCTCCTTTACCCC	EcoRV/SP6
*tfec*	F-TATAAAGACCGGACGGGGACAAC R-GCTCCTGGATTCGTAGCTGGA	EcoRV/SP6
*vax2*	F-TGACAGGAACGAACTTCGCTAGAC R-TCCGCTTCATCCGATTGGATGTTTGC	PmeI/T7

**Table 2:** Table of statistics

*p* value	Data structure	Type of test	95% confidence interval
a	Normal distribution	Unpaired *t* test	−3.213 to 1.076
b	Normal distribution	Unpaired *t* test	−2.735 to 0.3842
c	Normal distribution	Unpaired *t* test	−13.45 to 5.779
d	Normal distribution	Unpaired *t* test	−7.157 to 2.843
e	Normal distribution	Unpaired *t* test	−10.98 to 4.126
f	Normal distribution	Unpaired *t* test	−1.931 to 10.15
g	Normal distribution	Unpaired *t* test	−6.045 to 5.374
h	Normal distribution	Ordinary one-way ANOVA, Dunnett’s multiple comparison	0.2090 to 0.3762
i	Normal distribution	Ordinary one-way ANOVA, Dunnett’s multiple comparison	0.1456 to 0.3019
j	Non-normal distribution	χ^2^ contingency test	N/A
k	Non-normal distribution	Mann–Whitney *U* test	N/A
l	Non-normal distribution	Mann–Whitney *U* test	N/A
m	Normal distribution	Unpaired *t* test	−0.4634 to −0.2
n	Normal distribution	Unpaired *t* test	−0.4473 to −0.06999
o	Normal distribution	Unpaired *t* test	−9.210 to −2.790
p	Normal distribution	Ordinary one-way ANOVA, Dunnett’s multiple comparison	0.07807 to 0.2947
q	Normal distribution	Ordinary one-way ANOVA, Dunnett’s multiple comparison	0.1373 to 0.3642
r	Normal distribution	Ordinary one-way ANOVA, Dunnett’s multiple comparison	26.09 to 56.69
s	Normal distribution	Ordinary one-way ANOVA, Dunnett’s multiple comparison	14.00 to 45.54

### Confocal time-lapse imaging

For *in vivo* imaging, 12-ss embryos were dechorionated and mounted (dorsal head down at 12 ss) in 0.8% low melting point agarose (Invitrogen) in 1× E3 medium, then covered with 1× E3 containing 0.16 mg/ml tricaine (Research Organics). A stage heater was used to keep embryos at 29°C. Images were acquired by using a Zeiss LSM700 confocal microscope and Zen Black software (Zeiss): 24–30 *z*-sections, 3- to 5-μm *z*-step, 5-min intervals, 8- to 12-h time frame, 20× objective. NIH ImageJ (Schneider et al., 2012) software was used to manually track cells over time. Embryos that moved significantly in the recording period were not used for analysis.

### Eye explant cultures

Wild-type *Tg(tfec:EGFP)* eyes were dissected out at the 18 ss and plated on 0.01% poly L-lysine (Sigma) plus 25 μg/ml Fibronectin (Sigma) coated slides and grown in 1× MBS (88 mm NaCl, 1 mm KCl, 1 mm MgSO_4_, 5 mm HEPES, pH 7.8, and 2.5 mm NaHCO_3_) containing 10 μg/ml gentamycin (Sigma), with or without 300 ng/ml recombinant mouse Sema6d-Fc chimera (R&D Systems). Explants were cultured at 28°C for 20 h then live imaged on a Zeiss 40 CFL compound fluorescent microscope and, in a blinded fashion, both the number of *tfec*:EGFP+ cells that had exited the explants, and the percent coverage of the eye by RPE-GFP+ signal, quantified.

### Imaging of fixed tissue

Whole embryos were imaged in 3% methylcellulose using a Zeiss Stemi SV 11 microscope with an AxioCam HRc (Zeiss) camera and AxioVision (Zeiss) software. For sectioned samples, embryos were dehydrated with an ethanol work-up and embedded in JB4 medium (Polysciences Inc.) and 7-μm-thick sections were cut with a Leica microtome. Sections were collected on glass slides and mounted with a coverslip in Aquapolymount (Polysciences Inc.) and imaged using a Zeiss Axioplan2 microscope with an MRc camera (Zeiss) and AxioVision (Zeiss) software.

### Adobe Photoshop and Illustrator suites

Adobe CS5 Photoshop was used to crop and adjust brightness of images. Adobe CS5 Illustrator was used to compile and annotate data photographs as well as generate illustrations.

### Experimental design and statistical analyses (Table 2)

For characterization of the *sema6d* mutants we employed non-random experimental design statistical methods. For *plxna1* knock-down and pharmacological manipulations (Sema6d-Fc and dasatanib) we employed random experimental design statistical methods. Statistical analysis was performed by using Prism8 software (GraphPad). For analysis of mutants, sample size calculations were not performed because we had no prediction as to the likely size of effects to use in power calculations. Wild-type and mutant embryos were chosen randomly from clutches. All data were tested for normality by using the Shapiro–Wilk test. When the data were normally distributed, unpaired, two-tailed *t* tests were used to compare data between two samples, and a one-way ANOVA with *post hoc* test used for multiple comparisons. For the time lapse analysis, we used a non-parametric Mann–Whitney *U* test as sample sizes were too small to determine normality. In the text, we indicate the number of the number of biological replicates (*n*) and the number of independent replicates (*N*). Analysis of whole-mount RNA ISH embryos and quantitative measurements (except for time-lapse imaging cell speeds) were performed with the researcher blinded to genotype or condition.

## Results

### *sema6d* is expressed by neural retina progenitors and its receptor, *plxna1*, by adjacent RPE progenitors

We investigated the expression of genes encoding transmembrane Sema6 proteins in early zebrafish eye development (data not shown), which suggested possible roles for Sema6s in the control of eye progenitor movements. For instance, *sema6a* is expressed in the early eye vesicles as they evaginate bilaterally from the forebrain and elongate, and is required for proper entry of progenitors in a spatially regulated manner into the eye vesicles (Ebert et al., 2014). Here, we explore a role for Sema6d, whose mRNA is expressed by eye progenitors at the onset of optic cup morphogenesis. To understand the spatial and temporal expression of *sema6d*, we performed whole-mount RNA ISH with antisense riboprobes for *sema6d* and its receptor, *plxna1* (Toyofuku et al., 2004a,b), on zebrafish embryos ranging from the 8 ss to 24 hpf. Of note, there are two *plxna1* genes in zebrafish that are encoded on separate chromosomes. We refer to the *plxna1a* gene as that encoded on *Danio rerio* chromosome 23 (NCBI reference sequence XM_017353686.2), and to *plxna1b* as that encoded on chromosome 6 (NCBI reference sequence NM_001110010.1).

**Figure 1.**
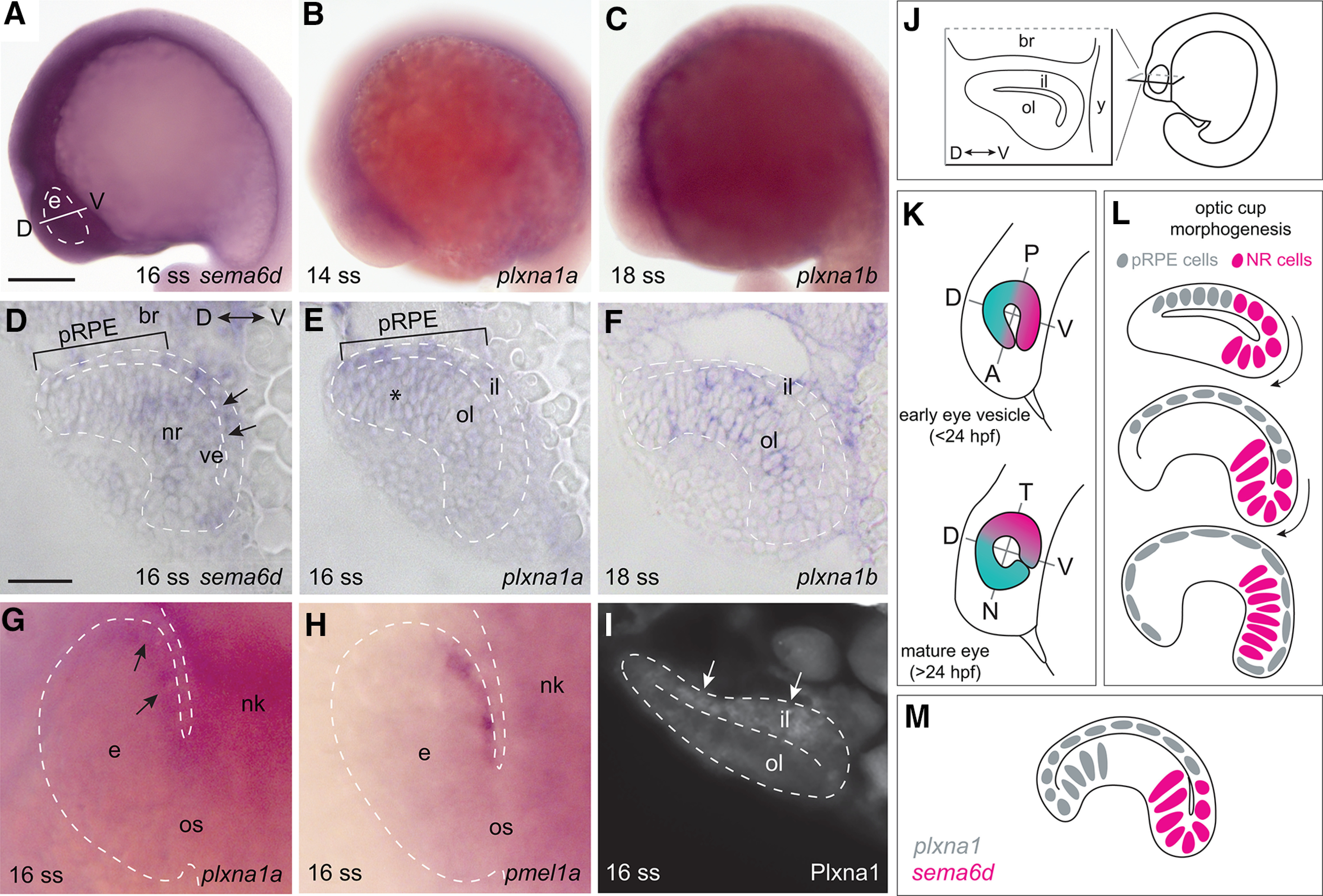
*sema6d* and *plxna1a* are expressed in complementary domains in the early eye vesicle. ***A***, Lateral view of a 16-ss zebrafish embryo shows *sema6d* transcript in the eye vesicle, and regions of the head and trunk. ***B***, ***C***, Embryos processed for RNA ISH reveal transcript present in the optic vesicle at the 14-ss (*plxna1a*) and 18-ss (*plxna1b*) stage. ***D–F***, Transverse sections (line in ***A***) through the brain and eye. *sema6d* transcript is expressed in the ventral (future temporal) domain of the inner eye vesicle leaflet (arrows) and neural retina (outer leaflet), but is absent from the dorsal (future nasal), presumptive RPE progenitor (pRPE) domain (bar; ***D***). *plxna1a* mRNA is present in the pRPE domain (bar) and faintly in the dorsal neural retina (asterisk), but absent from the ventral inner and outer eye vesicle leaflet (***E***). *plxna1b* is expressed in scattered cells of the outer leaflet of the developing optic cup (***F***). ***G***, ***H***, Eye vesicle (viewed dorsally) at the 16 ss processed for whole-mount ISH with antisense riboprobes for *plxna1a* (arrows in ***G***) or the RPE marker, *pmel1a* (***H***). ***I***, Plxna1-like immunoreactivity is present at the 16 ss in the pRPE domain (arrows). ***J–L***, Schematics of the eye vesicle (***J***), the early embryo axes with respect to the eye (***K***), and optic cup morphogenesis (***L***); note that as the eye rotates alongside brain development, early ventral retina tissue (pink) becomes mature temporal tissue. ***M***, Schematic of *plxna1a* and *sema6d* mRNA expression in the 16-ss eye vesicle. Scale bars: 300 μm (***A***) and 50 μm (***D***). br: brain, D: dorsal, e: eye, il: inner leaflet, N: nasal, nk: neural keel, nr: neural retina, ol: outer leaflet, os: optic stalk, pRPE: presumptive RPE, RPE: retinal pigment epithelium, T: temporal, V: ventral, ve: ventricle.

The timing of optic cup morphogenesis in zebrafish is well described; eye vesicles evaginate from the neural keel as early as the 4 ss (Ivanovitch et al., 2013), and invaginate around the forming lens starting at about the 17 ss (Li et al., 2000), with optic cup morphogenesis mostly complete by 24 hpf (Kwan et al., 2012; Heermann et al., 2015). Transcript for *sema6d* was first detected at approximately the 16 ss, with expression in the eye vesicle, brain and ventrally along the anteroposterior axis of the embryo (Fig. 1*A*). In plastic transverse sections through the eye, *sema6d* was expressed more highly in the ventral portion of the inner (Fig. 1*D*, arrows; future temporal retina) and outer leaflets of the eye vesicle. Notably, *sema6d* mRNA was absent from the presumptive RPE domain, which sits within the dorsal inner leaflet (Fig. 1*D*, bar). By 24 hpf, staining for *sema6d* transcript was localized specifically to the temporal optic cup (data not shown). Of note, between 16 and 24 hpf, eye morphogenesis alongside flexing of the brain changes the early dorsoventral axis of the retina to that of nasotemporal, such that “early ventral” becomes “mature temporal” tissue, and “early dorsal” becomes “mature nasal” tissue (Fig. 1*K*). In dorsal whole mount, transcript for *plxna1a* was detected at approximately the 14 ss in the neural keel (Fig. 1*B*), and in the presumptive RPE domain (shown at the 16 ss; Fig. 1*G*, arrows), as indicated by the RPE marker *pmel1a* (Cechmanek and McFarlane, 2017; Fig. 1*H*). This expression was confirmed in section, where *plxna1a* ISH signal and Plxna1-like immunoreactivity, with a rabbit polyclonal antibody against human PLXNA1, were visible in the presumptive RPE progenitor domain of the dorsal inner leaflet of the eye vesicle (Fig. 1*E*,*I*), with faint signal in the dorsal (future nasal) neural retina (Fig. 1*E*, asterisk). By 24 hpf, *plxna1a* transcript is only weakly expressed by the RPE (data not shown). RNA ISH for the *plxna1a* homolog, *plxna1b*, revealed minimal expression at the 14 ss (data not shown), and transcript in the presumptive neural retina in a spattering of cells at 18 hpf (Fig. 1*C*,*F*). In summary, the expression of *sema6d* and *plxna1a* in complementary domains over the period of optic cup morphogenesis (Fig. 1*M*) suggests they may interact to control the cell movements that result in the production of an optic cup.

### Temporal optic cup shows defects in sema6d mutants

**Figure 2.**
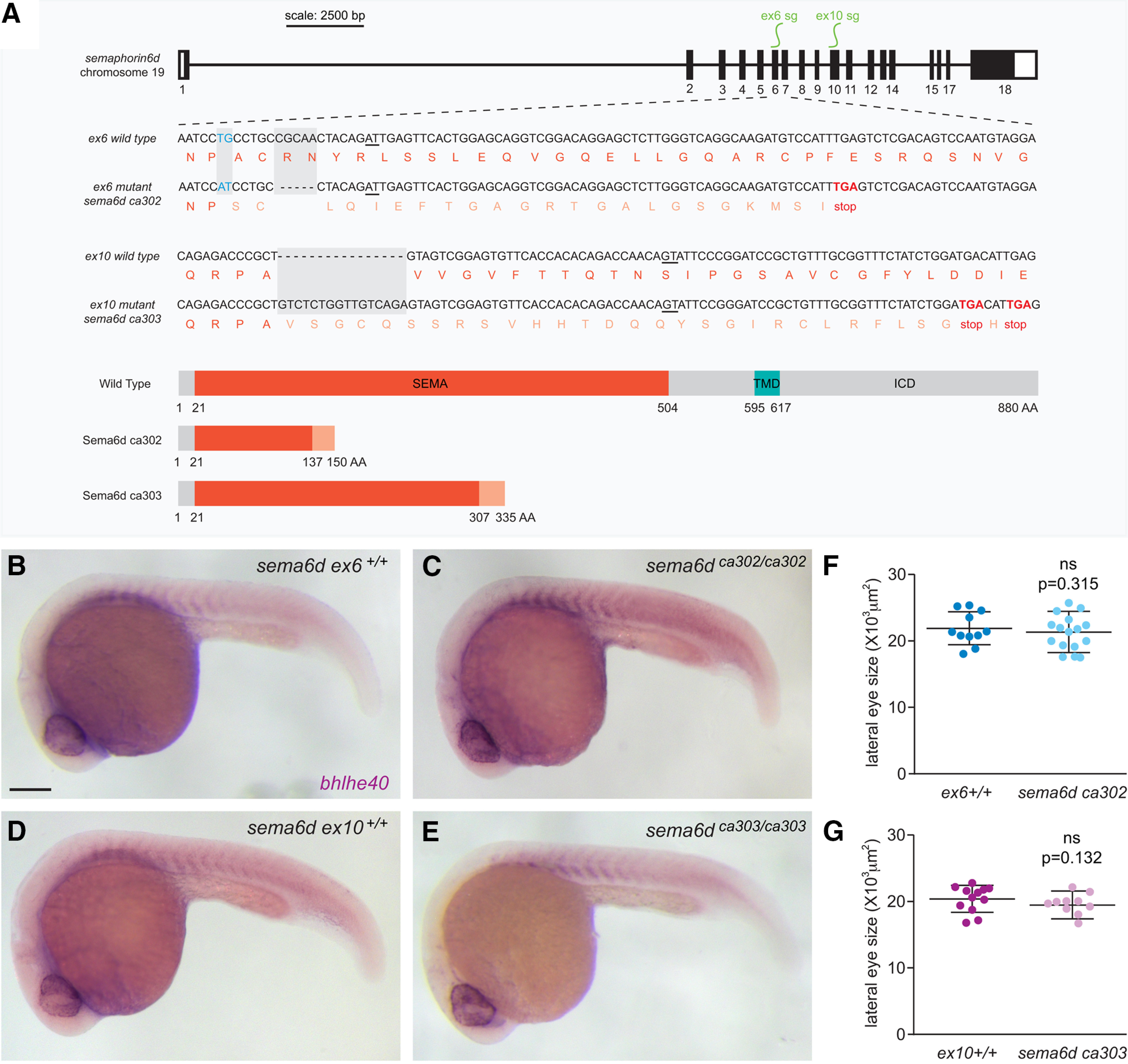
*sema6d* mutants do not display gross morphologic defects. ***A***, Schematic of the two *sema6d* mutant alleles; *sema6d^ca302^* mutants contain a five-base pair deletion and two-base pair mismatch in exon6, and *sema6d^ca303^* mutants contain a 16-base pair insertion in exon10, both predicted to generate proteins that truncate prematurely within the SEMA domain and result in the loss of the transmembrane domain (TMD) and the intracellular domain (ICD). ***B–E***, Lateral views of whole-mount embryos processed for *bhlhe40* ISH, revealing normal gross embryonic morphology and somite labeling in both mutant alleles with respect to their wild-type siblings at 24 hpf. ***F***, ***G***, Eye area measured from lateral images of *sema6d* mutants and their respective wild-type siblings at 24 hpf [unpaired *t* test, df = 26 (***F***), df = 21 (***G***); error bars are SD]. Scale bar: 200 μm.

To study Sema6d loss-of-function *in vivo* we used CRISPR/Cas9 gene editing techniques (Hwang et al., 2013; Auer et al., 2014; Gagnon et al., 2014; Montague et al., 2014) to generate two mutant fish lines. Suitable sgRNAs could not be targeted to exons upstream of the Sema domain, and so we generated sgRNAs to target exons 6 and 10. The *sema6d* exon6 (*sema6d^ca302^*) and exon10 (*sema6d^ca303^*) mutant lines are predicted to generate truncated proteins of 137 and 307 amino acids in length, respectively. The truncated proteins would both lack the transmembrane and intracellular domains (Fig. 2*A*), and contain an incomplete Sema domain, which is required in whole for homodimerization and binding of Plxna1 to induce forward signaling (Janssen et al., 2010; Nogi et al., 2010).

Initial analyses indicated that the gross development of both *sema6d* mutant lines (*sema6d^ca302^*, *sema6d^ca303^*) and their wild-type siblings (*sema6d ex6^+/+^*, *sema6d ex10^+/+^*, respectively) was qualitatively similar. Embryos processed at 24 hpf for RNA ISH against *bhlhe40* (an early RPE and somite marker; Cechmanek and McFarlane, 2017), to visualize gross eye anatomy and size, had normal body axes and patterned somites (Fig. 2*B–E*). Further, measurements of the lateral areas of *sema6d^ca302^* [*N* = 2; *sema6d ex6^+/+^* 21.83 × 10^3^ ± 2.5 × 10^3^ μm^2^ (SD; here and throughout paper unless otherwise indicated) *n *=* *11, *sema6d^ca302^* 20.76 × 10^3^ ± 2.8 × 10^3^ μm^2^ *n *=* *17; *p*^a^ = 0.315, unpaired *t* test, df = 26] and *sema6d^ca303^* (*N* = 2; *sema6d ex10^+/+^* 20.30 × 10^3^ ± 1.9 × 10^3^ μm^2^ *n *=* *13, *sema6d^ca303^* 19.12 × 10^3^ ± 1.6 × 10^3^ μm^2^ *n *=* *10; *p*^b^ = 0.132, unpaired *t* test, df = 21) eyes were not significantly different from those of their wild-type siblings (Fig. 2*F*,*G*). For subsequent analysis, we focused mainly on the *sema6d^ca302^* line, using the *sema6d^ca303^* line to confirm key findings. Of note, RT-qPCR showed that *sema6d* levels are downregulated in 48-hpf *sema6d^ca302^* embryos (0.3 ± 0.06-fold change, *N* = 3 independent experiments; average of three technical replicates/experiment), suggesting the occurrence of nonsense-mediated mRNA decay (Miller and Pearce, 2014).

**Figure 3.**
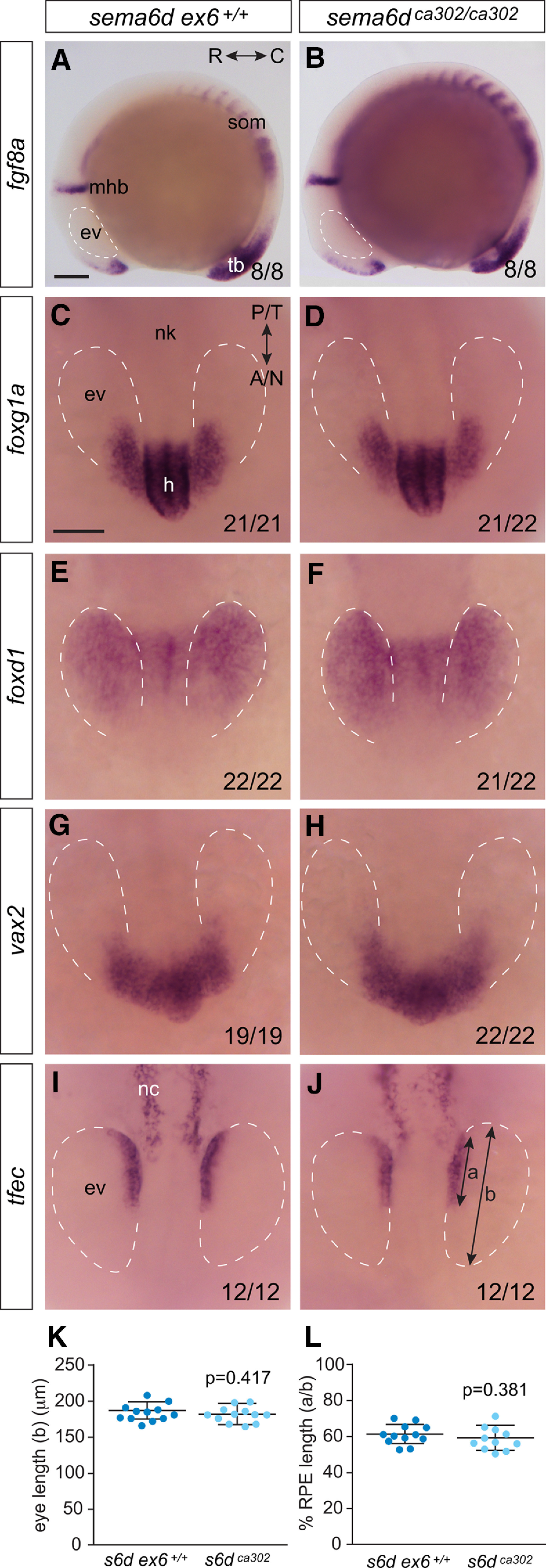
*sema6d* mutants display normal eye vesicle development and patterning before optic cup morphogenesis. Whole-mount RNA ISH for patterning genes indicates early development occurs normally in *sema6d* mutants (***B***, ***D***, ***F***, ***H***, ***J***) relative to wild-type siblings (***A***, **C**, ***E***, ***G***, ***I***). In the bottom right of panels are the number of embryos of the total analyzed that exhibited the normal wild-type expression pattern. ***A***, ***B***, *fgf8a* expression in 10-ss embryos viewed laterally. ***C–H***, Dorsal whole-mount images of the eye vesicles (dashed outlines) and neural keel of 12 ss processed by whole-mount ISH for the expression of early patterning genes. ***C***, ***D***, Early dorsal (future nasal) tissue with *foxg1a*. ***E***, ***F***, Early ventral (future temporal) tissue with *foxd1*. ***G***, ***H***, Early anterior (future ventral) tissue with *vax2*. ***I***, ***J***, RPE progenitors and neural crest cells express *tfec* at the appropriate time in wild-type siblings and *sema6d* mutants. ***K***, The average anteroposterior length of the eye vesicles. ***L***, Ratio (%) of RPE to eye vesicle anteroposterior lengths [bars a:b in ***J***; *p* values are unpaired *t* test, df = 22 (***K***), df = 21 (***L***), error bars are SD]. Scale bars: 100 μm. A: anterior, C: caudal, ev: eye vesicle, h: hypothalamus, mhb: mid-hindbrain boundary, N: nasal, nc: neural crest, nk: neural keel, P: posterior, R: rostral, som: somites, T: temporal, tb: tailbud.

Transcript for *sema6d* is not detected readily until the 14 ss, and thus, we expected that early embryogenesis and eye vesicle development should be unaffected in *sema6d* mutants. To verify that this was indeed the case, we assayed early development of the *sema6d* mutant embryo and eyes. *fibroblast growth factor-8* (*fgf8a*) expression at the 10 ss in the anterior forebrain, at the midbrain-hindbrain boundary, in the somites and in the tail bud argues that the general body plan is normal in *sema6d* mutants (Fig. 3*A*,*B*). When assessed in a blinded manner, the morphology, size (*N* = 2; *sema6d ex6^+/+^* 185.8 ± 11.9 μm, *n *=* *12; *sema6d^ca302^* 182.0 ± 10.8 μm, *n *=* *12; *p*^c^ = 0.417, unpaired *t* test, df = 22; Fig. 3*K*), and patterning of the early eye vesicles were also similar in *sema6d* mutants and their wild-type siblings (Fig. 3*C–H*). Bilateral eye vesicles evaginated from the forebrain and had elongated. Further, whole-mount ISH at the 12 ss revealed proper early eye patterning had occurred in both *sema6d* mutants and wild-type siblings; dorsal (future nasal) tissue was present and appropriately labeled with *foxg1a* (Picker et al., 2009; Hernández-Bejarano et al., 2015), ventral (future temporal) tissue likewise with *foxd1* (Picker et al., 2009; Hernández-Bejarano et al., 2015), and anterior (future ventral) tissue with *vax2* (French et al., 2009; Fig. 3*C–H*). RPE progenitors were specified, as assayed by *tfec* expression (Fig. 3*I*,*J*), and the RPE domain expanded anterowards in an appropriate manner (*N* = 2; wild-type 60.9 ± 5.5% coverage of eye vesicle by RPE, *n *=* *12; *sema6d^ca302^*58.8 ± 6.3%, *n *=* *12; *p*^d^ = 0.381, unpaired *t* test, df = 22; Fig. 3*L*; Cechmanek and McFarlane, 2017). Together, these data indicate early molecular and morphologic development of the eyes is normal in the *sema6d* mutants.

**Figure 4.**
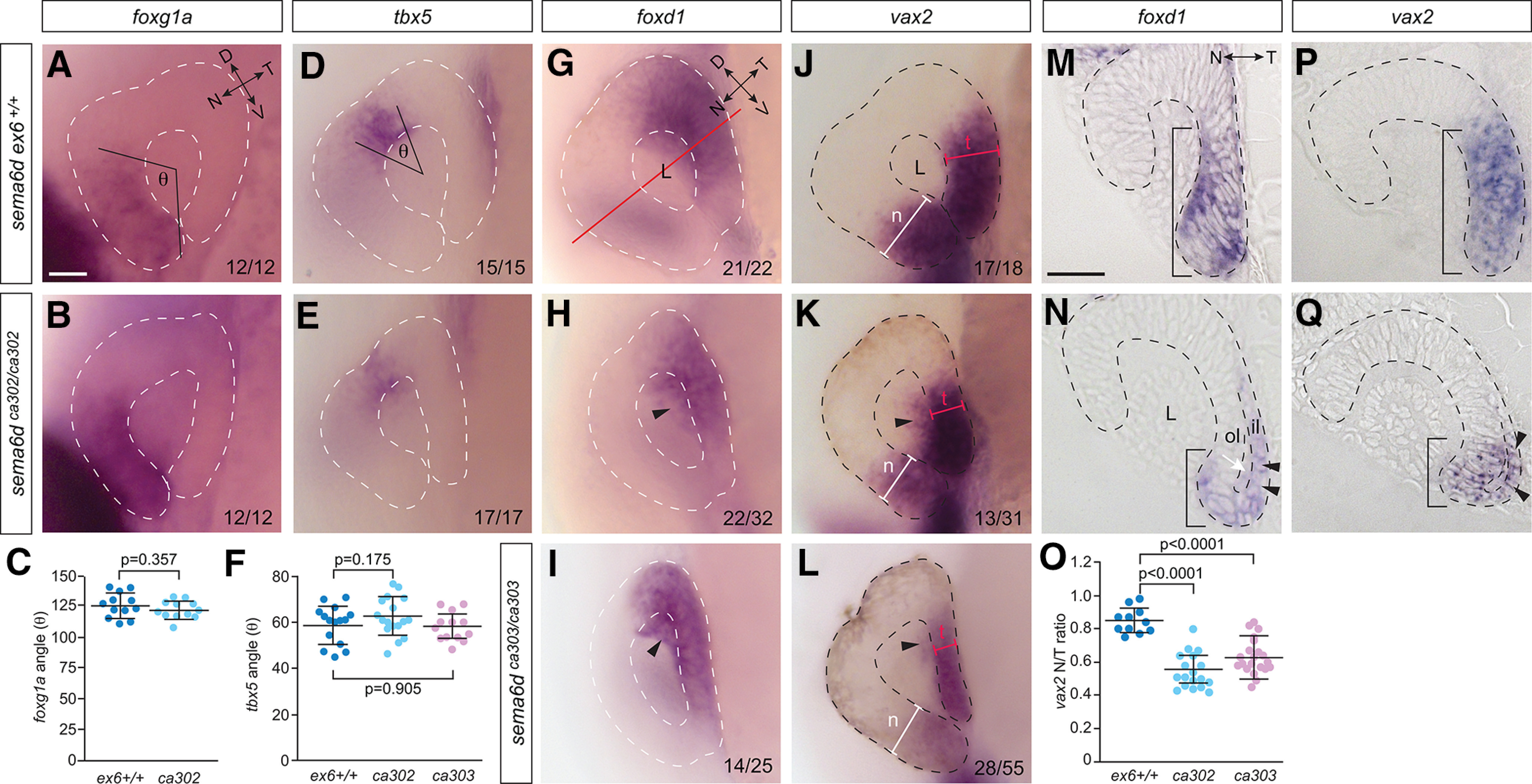
*sema6d* mutants display temporal neural retina defects post optic cup morphogenesis. ***A***, ***B***, ***D***, ***E***, Lateral images of 24-hpf optic cups processed for RNA ISH in wild-type (***A***, ***D***) and *sema6d* mutant (***B***, ***E***) embryos show similar expression of nasal (*foxg1a*) and dorsal (*tbx5*) markers. Of note, these domains derive from the dorsal and posterior eye vesicle, respectively. In the bottom right of panels are the number of embryos of the total analyzed that exhibited either a WT (***A***, ***B***, ***D***, ***E***, ***G***, ***J***) or a disrupted (***I,K,L***) expression pattern. ***C***, ***F***, The angle formed by the lateral edges of the *foxg1a* (***C***; *p* values are unpaired *t* test, df = 22, error bars are SD) and *tbx5* (***F***; *p* values are unpaired *t* test, df = 30 *ex6+/+* vs *ca302*, df = 24 *ex6+/+* vs *ca303*, error bars are SD) domains to the center of the lens (θ). **G–*L***, *foxd1* and *vax2* RNA ISH viewed in whole mount. The shape and size of temporal eye (early ventral) markers, *vax2* and *foxd1*, are disrupted in the *sema6d^302^* (***H***, ***K***) and *sema6d^303^* (***I***, ***L***) mutants as compared with wild-type siblings (***G***, ***J***). Black arrowheads point to aberrant *vax2* and *foxd1* label in the inner vesicle leaflet, seen through the depth of the eye in the transparent zebrafish embryo. ***M***, ***N***, ***P***, ***Q***, Transverse plastic sections (axis shown by red line in ***G***) through the central retina of 24-hpf wild-type sibling and *sema6d* mutant eyes processed for *foxd1* and *vax2* RNA ISH. Note in mutants a smaller (compare t in ***J–L*** and length of bars in ***M***, ***P*** vs ***N***, ***Q***) *foxd1/vax2* domain in the temporal neural retina, and an open ventricle as compared to wild-type (white arrow in ***N***). ***O***, Blinded quantitation of the ratio of the width of *vax2*+ domain in temporal versus nasal optic cup (t and n in ***J–L***), which captures the redistribution of *vax2* expression in *sema6d* mutants (*p* values are one-way ANOVA, Dunnett’s multiple comparisons test, error bars are SD, df = 53). Scale bars: 50 μm (***A***, ***M***). D: dorsal, il: inner leaflet, L: lens, N: nasal, ol: outer leaflet, T: temporal, V: ventral.

*sema6d* transcript appears just before optic cup morphogenesis. Therefore, we next asked whether Sema6d was involved in optic cup formation after the 14 ss. First, we assayed embryos at 24 hpf to see whether patterning of the optic cup occurred normally in the mutants. We processed embryos for RNA ISH against markers of the nasal (early dorsal; *foxg1a*), dorsal (early posterior; *tbx5a*; French et al., 2009), temporal (early ventral; *foxd1*), and ventral (early anterior; *vax2*) mature neural retina (Fig. 4). Whole-mount eyes were imaged and assessed in a blinded manner. *foxg1a* and *tbx5a* were expressed similarly in the nasal and dorsal eye, respectively, of mutant embryos and their wild-type siblings (Fig. 4*A*,*B*,*D*,*E*), as indicated by the similarity in the angles that span the expression domains across genotypes (*foxg1a* angle: *sema6d ex6^+/+^* vs *sema6d^ca302^ p*^e^ = 0.357, unpaired *t* test, *tbx5a* angle: *sema6d ex6^+/+^* vs *sema6d^ca302^ p*^f^ = 0.175, unpaired *t* test, *sema6d ex6^+/+^* vs *sema6d^ca303^ p*^g^ = 0.905, unpaired *t* test; Fig. 4*C*,*F*). In contrast, the gross appearance of *foxd1* and *vax2* staining suggested a defect in development of the temporal eye (Fig. 4*G–L*): temporal domains of expression were aberrant in size (red bars), shape, and pattern (black arrowheads). Importantly, defects presented in both mutant alleles and in the tissue that expresses *sema6d* during optic cup morphogenesis; the ventral region of the early eye vesicle that contains the neural progenitors that go on to populate the temporal neural retina.

To better understand the defect in the temporal eye at 24 hpf, 7-μm-thick transverse sections were made of wild-type and mutant whole-mount *foxd1+* and *vax2+* embryos (Fig. 4*M*,*N*,*P*,*Q*). During optic cup morphogenesis, the neural retinal progenitors of the ventral inner leaflet of the eye vesicle move around the distal rim of the eye into the outer leaflet to become the temporal neural retina of the 24-hpf optic cup (Picker et al., 2009; Kwan et al., 2012; Fig. 1*K*,*L*). Concurrently, the RPE cells of the dorsal eye vesicle are stretched in both ventral and dorsal directions and end up as a single cell layer thick epithelium that lines the back of the neural retina (Heermann et al., 2015; Cechmanek and McFarlane, 2017). In wild-type siblings, this process of morphogenesis occurred normally, so that at 24 hpf the *foxd1+* and *vax2+* domains sat within the neural retina (Fig. 4*M*,*P*, bar). Further, labeling of these markers was absent from the thinned inner leaflet, now comprising the RPE. In mutant eyes (Fig. 4*N*,*Q*), fewer *foxd1*+ and *vax2+* cells sat within the neural retina of the outer leaflet (bars), and a significant proportion of cells aberrantly populated the inner leaflet (arrowheads). To quantitate the temporal eye phenotype, we compared the width of the *vax2* ISH domain in the temporal versus nasal eye, in lateral whole-mount images (Fig. 4*J–L*,O): much of the temporal *vax2* signal was found aberrantly in the inner eye vesicle leaflet of mutants (Fig. 4*K*,*L*, arrowheads), hidden behind the portion of the *vax2* domain in the outer leaflet (Fig. *4K*,*L*, red bar), so that in whole mount the outer leaflet *vax2+* temporal domain was often more narrow than that observed in wild-type. As such, the ratio of the temporal versus nasal width of the *vax2* domain was decreased significantly in *sema6d* mutant embryos as compared with wild-type siblings (*N* = 2; *ex6^+/+^* 0.85 ± 0.08, *n *=* *11; *sema6d^ca302^*0.56 ± 0.1, *n *=* *18, *p*^h^ < 0.0001; *sema6d^ca303^*0.62 ± 0.1, *n *=* *27; π < 0.0001, one-way ANOVA, Dunnett’s multiple comparisons test, df = 53; Fig. 4*O*). The specificity of the defect to temporal (early ventral) and not nasal (early dorsal) eye tissue agrees with the localization of *sema6d* mRNA to neural progenitors of only the ventral domain of the early eye vesicle.

### RPE temporal expansion is disrupted in *sema6d* mutant embryos

**Figure 5.**
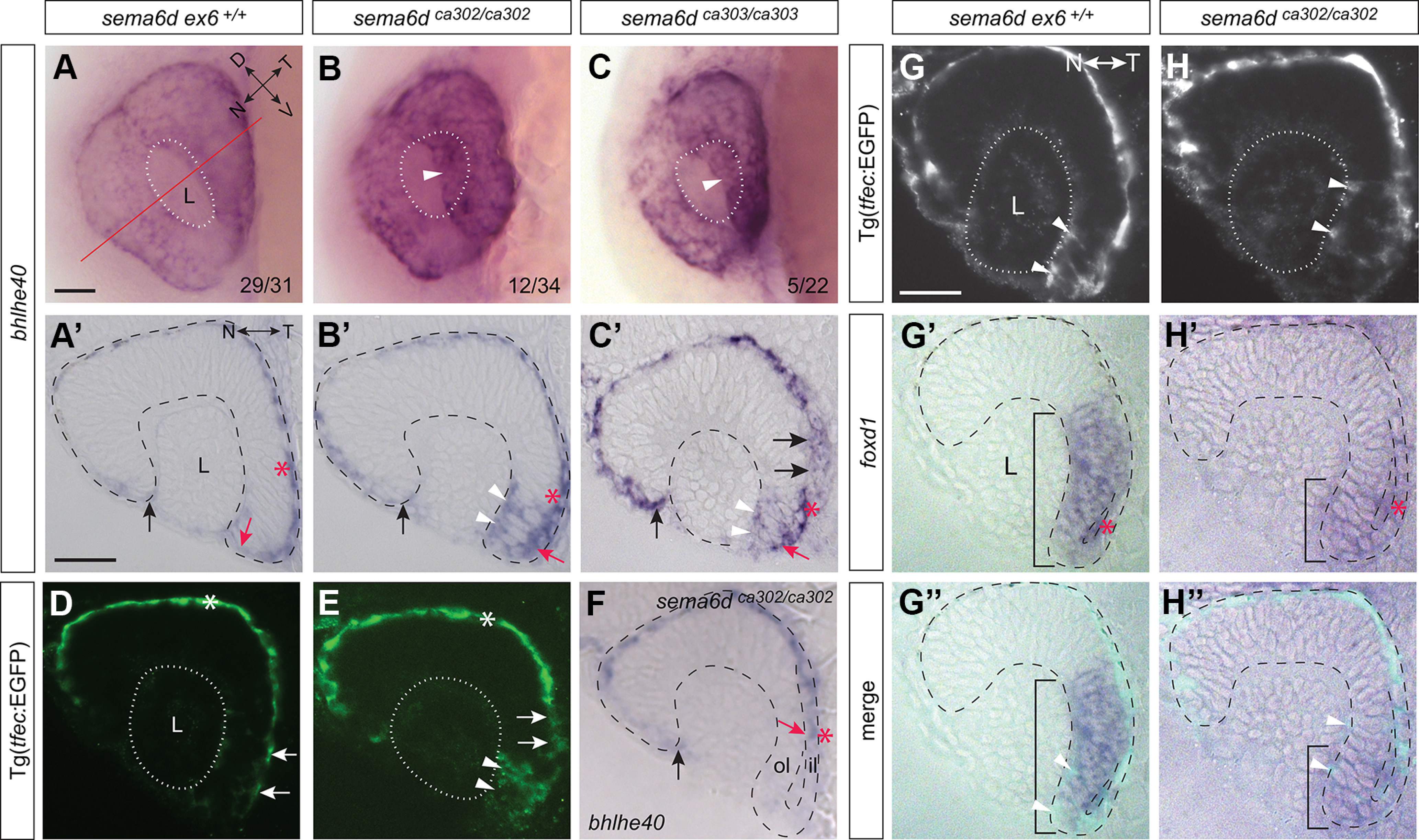
Disrupted RPE morphogenesis in *sema6d* mutants. ***A–C***, Morphogenesis of the RPE is disrupted in *sema6d^ca302^* and *sema6d^ca303^* eyes as compared with wild-type, with mutants displaying ectopic *bhlhe40* staining apparent through the transparent lens (white arrowhead in ***B***, ***C***). In the bottom right of panels are the number of embryos of the total analyzed that exhibited either a WT (***A***) or a disrupted (***B***, ***C***) expression pattern. ***A’–C’***, ***F***, Transverse sections reveal full expansion of the *bhlhe40+* RPE to abut the nasal lens (black arrows), while the RPE of the temporal eye fails to reach the lens in mutants (compare red arrows). RPE cells are expanded in the inner leaflet of wild-type embryos, but cuboidal in mutants (compare red asterisks). In most mutants the *bhlhe40*+ RPE not only failed to abut the lens, but *bhlhe40* was ectopically expressed in the lateral portion of the temporal neural retina (white arrowheads in ***B’***, ***C’***), while in some *sema6d* mutants, *bhlhe40* expression was restricted entirely to the inner eye vesicle leaflet (red arrow in ***F***). ***D***, ***E***, Wild-type (***D***) and *sema6d^ca302^* mutant (***E***) embryos bred on a *Tg(tfec:EGFP)* background to label RPE progenitors. Mutants display ectopic EGFP expression in the temporal neural retina (arrowheads in ***E***) and EGFP+ RPE cells in the temporal (early ventral) inner leaflet are cuboidal and not extended as in wild-type (compare arrows in ***D***, ***E***). ***G***, ***H***, Transverse sections of 22-hpf eyes of wild-type (***G–G’’***) and *sema6d^ca302^* (***H–H’’***) mutant embryos on a *Tg(tfec:EGFP)* background that were processed for GFP immunohistochemistry (***G***, ***H***) and RNA ISH for the temporal neural retina marker *foxd1* (***G’***, ***H’***). Blended images (***G’’***, ***H’’***). EGFP+ cells that co-express *foxd1* are present in the distal temporal neural retina of both wild-type and mutant eyes (arrowheads in ***G***, ***G’’***, ***H***, ***H’’***). A few *foxd1*+ cells in the wild-type inner leaflet (asterisk in ***G’***) have not yet moved around the distal rim, with many more present in the mutant inner leaflet (asterisk in ***H’***). Scale bars: 50 μm (***A***, ***A’***). D: dorsal, il: inner leaflet, L: lens, N: nasal, ol: outer leaflet, T: temporal, V: ventral.

Given the disruption of the normal redistribution of neural eye progenitors from the inner leaflet of the eye vesicle to the temporal neural retina, we asked whether the neighboring RPE cells were affected in the *sema6d* mutant by assaying for expression of the RPE markers *bhlhe40* and *tfec* (Cechmanek and McFarlane, 2017). In the eyes of whole-mount wild-type embryos at 24 hpf, *bhlhe40*+ RPE cells abutted the lens (Fig. 5*A*). In both mutant alleles, however, *bhlhe40* signal was often seen through the transparent zebrafish lens where in wild-type eyes signal was not observed (Fig. 5*B*,*C*, arrowhead). The difference between wild-type and *sema6d* mutants was particularly evident in transverse sections (Fig. 5*A’–C’*,*F*). In both wild-type and *sema6d* mutant embryos, *bhlhe40+* RPE cells stretched normally toward the nasal lens (black arrows). In sections of the occasional mutant (11%, *n *= 18 eyes), we found as expected, a complete failure of *bhlhe40+* RPE cells to extend around the temporal optic cup (early ventral), with the *bhlhe40* ISH label present only within the inner eye vesicle leaflet (Fig. 5*F*, red arrow). More common (72%, *n *= 18 eyes), however, was the unexpected presence of ectopic *bhlhe40+* cells present in the distal portion of the temporal neural retina of mutants (Fig. 5*B’*,*C’*, arrowheads). Importantly, these phenotypes were present in both mutant alleles (wild-type, normal RPE morphogenesis *n *=* *12/12; aberrant morphogenesis, *sema6d^ca302^ n = *12/34 and *sema6d^ca303^ n *=* *5/22). Unlike the expanded RPE cells of wild-type eyes, in both *sema6d* mutant alleles RPE cells adjacent to the temporal optic cup were often cuboidal in appearance (compare Fig. 5*A’–C’*,*F’*, red asterisk). We crossed the *sema6d^ca302^* line onto a *Tg(tfec:EGFP)* reporter line (Miesfeld et al., 2015) that we regenerated, to examine EGFP+ RPE cells in transverse sections at 24 hpf (Fig. 5*D*,*E*). In agreement with the *bhlhe40* data, a thin monolayer RPE backed the central retina of both wild-type and mutant siblings (Fig. 5*D*,*E*, asterisk). Extension of the RPE around the temporal (early ventral) retina, however, only occurred in wild-type eyes (Fig. 5*D*, arrows). In the mutants, weak EGFP (*tfec*) label was present ectopically within the neural retina (Fig. 5*E*, arrowheads), and cells within the inner leaflet were often cuboidal in shape (Fig. 5*E*, arrows).

The ectopic *bhlhe40+/tfec+* cells within the distal temporal neural retina indicated that progenitors that expressed the temporal retinal markers *vax2* and *foxd1* also expressed an RPE marker (compare Figs. 4*N*,*Q* and 5*B’*,*C’*,*E*). While initially surprising, we found that it was normal for temporal neural retina progenitors to transiently co-express neural and RPE markers in wild-type eyes younger by a few hours (Fig. 5*G–G’’*, arrowheads). Indeed, in both wild-type and *sema6d^ca302^ Tg(tfec:EGFP)* 22-hpf eyes, *foxd1+* temporal progenitors were weakly EGFP-positive (Fig. 5*G*,*H*, arrowheads), indicating that these cells either expressed *tfec*, or recently turned off *tfec* expression. By 24 hpf, when optic cup morphogenesis was complete, wild-type siblings did not express *bhlhe40* or the *tfec*:EGFP reporter in cells within the temporal domain of the neural retina, instead only the flattened RPE cells expressed label (Fig. 5*D*). The wild-type data suggest that a population of phenotypically plastic neural retina progenitors, at the border between temporal neural and RPE domains, transiently express both RPE and temporal neural retina markers, before halting expression of RPE-related transcription factors. In mutants, these *foxd1+/vax2+* neural progenitors (Fig. 4*N*,*Q*), both within the outer leaflet and those aberrantly located in the inner leaflet, appeared to fail to turn off RPE specific transcription factors (Fig. 5*B’,E*, arrowheads). The result was that the temporal neural progenitors continued to co-express RPE markers inappropriately at 24 hpf.

**Figure 6.**
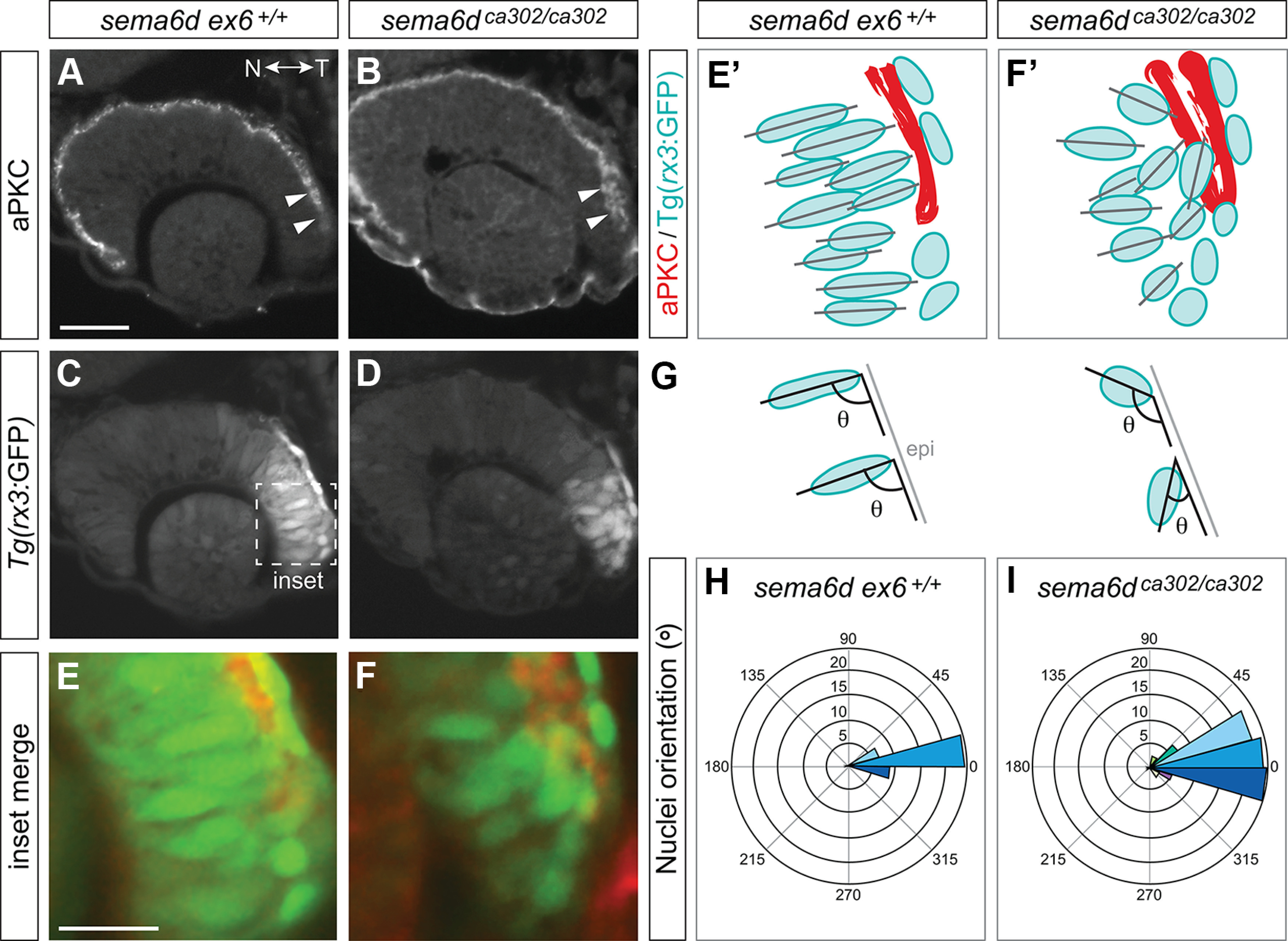
Temporal neural retina is disorganized in *sema6d* mutants. ***A–F***, Immunohistochemistry for the apical marker aPKC (***A***, ***B***) of wild-type and *sema6d* mutant (***C***, ***D***) *Tg(rx3:GFP)* embryos. Magnified view of merge of labels in temporal eye (boxed area in ***C***; ***E*, *F***). ***E’–F’***, Schematic of ***E***, ***F*** showing ventricle as marked by aPKC immunoreactivity (red), labeled GFP (blue) cells, and their orientation (black lines). In *sema6d* mutants, there is a failure of the temporal ventricle to seal (arrowheads in ***B***) and disorganization of temporal neural retina cells (***D***, ***F***, ***F’***). ***G***, ***H***, Distributions of the angles made by the long axis of DAPI-labeled nuclei with the basal temporal retinal neuroepithelium at 24 hpf. Average of the distributions in mutants (***I***, *n *=* *12 embryos) and wild-type sibling (***H***, *n *=* *13 embryos) are significantly different (*p* < 0.0001, χ^2^ contingency test). Circles indicate the average numbers of nuclei found in each 15° bin. Scale bars: 50 μm (***A***) and 20 μm (***E***). N: nasal, T: temporal.

Potentially as a consequence of cells in the temporal neural retina of *sema6d* mutants inappropriately expressing RPE-specific genes, the morphology of these cells was affected. *sema6d^ca302^* and their respective *sema6d ex6^+/+^* siblings were crossed onto a *Tg(rx3:GFP)* background, where initially all eye progenitors express GFP. By 24 hpf the cells in the temporal eye expressed higher levels of GFP, allowing us to look at cell morphology. Eye sections at 24 hpf were immunolabeled with an antibody that recognizes the apically located tight junction associated protein atypical protein kinase C (aPKC; Suzuki et al., 2001; Fig. 6*A*,*B*). In transverse microtome sections, the ventricle of the temporal optic cup in affected *sema6d* mutants did not exhibit the “zippering-up” that occurs in wild-type siblings (compare Fig. 6*A*,*B*, arrowheads). The aPKC label of the neural retina/RPE border in the nasal eye, however, appeared unaffected. Also of note was that while wild-type embryos had GFP+ cells in the temporal neural retina with typical elongated morphologies and radial alignments (Fig. 6*C*,*E*,*E’*), the progenitors in this domain in *sema6d^ca302^* eyes appeared disorganized and were not all aligned radially (Fig. 6*D*,*F*,*F’*). Indeed, the angles the DAPI-labeled nuclei make with the basal epithelial surface (Fig. 6*G*) were significantly more broadly distributed in the *sema6d^ca302^* mutants as compared with their wild-type siblings [*ex6^+/+^ n* = 13 embryos (174 nuclei), *sema6d^ca302^ n* = 12 embryos (170 nuclei); *p*^j^ < 0.0001, χ^2^ contingency test; Fig. 6*H*,*I*].

### Temporal eye defects caused by loss of Sema6d signaling are because of aberrant progenitor cell movements

**Figure 7.**
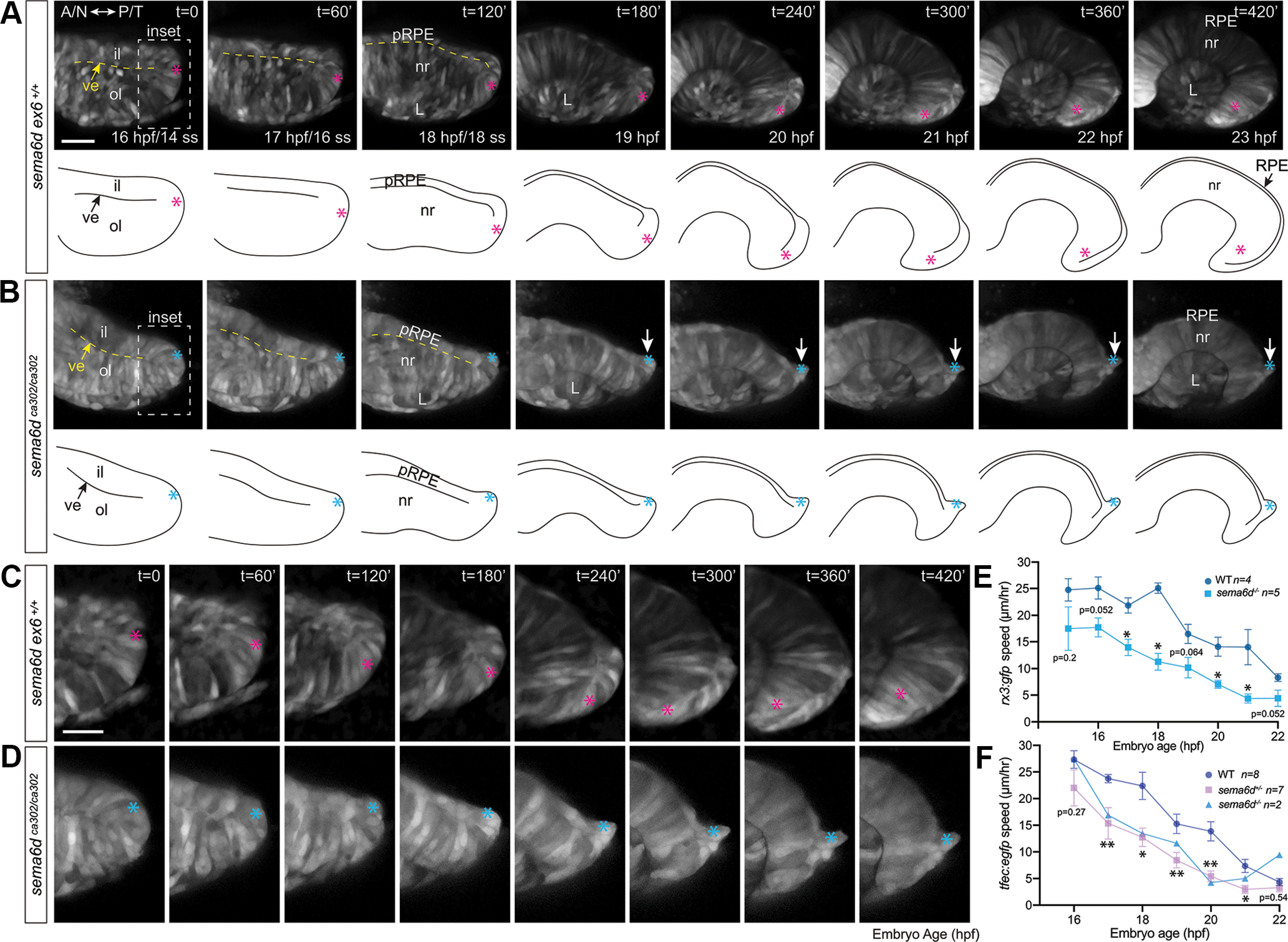
Ventral inner leaflet cells fail to move appropriately around the distal rim during optic cup morphogenesis. ***A***, ***B***, Confocal optical sections, and their corresponding schematics, acquired from the nasal/temporal plane between the 14 ss and 23 hpf of wild-type and *sema6d* mutant *Tg(rx3:GFP)* eyes (labels all eye progenitors). Dotted yellow line(s) indicates the separation between the inner and outer leaflets. ***C***, ***D***, Higher magnification of inset boxes (early ventral/future temporal retina) in ***A***, ***B***, respectively. In wild type (***A***), ventral progenitors move around the distal rim of the optic cup and come to reside in the temporal neural retina at 23 hpf. In contrast, *sema6d* mutants display temporal defects (follow arrow in ***B***). While a ventral inner leaflet cells in wild type (***C***) moves around the distal rim (pink asterisk), a corresponding cell in the *sema6d* mutant (***D***) begins to move toward the distal rim, but stalls and, together with other progenitors, protrudes from the inner leaflet (blue asterisk). ***E***, ***F***, Mean of the speed of individual cells in each wild-type and *sema6d* mutant embryo tracked over the period of optic cup morphogenesis. Tracked were ventral neural progenitors located at the distal eye vesicle tip in the *Tg(rx3:GFP)* background (***E***) and brightly EGFP labeled presumptive RPE cells in the *Tg(tfec:EGFP)* background (***F***). In both reporter lines, *sema6d* mutant cells move more slowly than their wild-type counterparts, and stall at ∼18–20 hpf. *n*s are number of embryos assayed (2–7 cells/embryo), and error bars are SEM. Mean speeds at individual time points were compared statistically between wild-type and heterozygous embryos by a Mann–Whitney *U* test (*p* < 0.05; ***E***, ***F***). A: anterior, il: inner leaflet, L: lens, N: nasal, nr: neural retina, ol: outer leaflet, P: posterior, pRPE: presumptive RPE, RPE: retinal pigment epithelium, T: temporal, ve: ventricle.

To understand the cell behaviours that lead to temporal disorganization in mutant eyes, we embedded *Tg(rx3:GFP)* and *Tg(tfec:EGFP)* wild-type and *sema6d* mutant embryos in low melting temperature agarose and imaged them over the period of optic cup morphogenesis by confocal microscopy (approximately from the 14 ss to 23 hpf). We used the *Tg(rx3:GFP)* background to label neural retina progenitors, while on the *Tg(tfec:EGFP)* background we identified presumptive RPE cells as the brightest EGFP+ cells, which we found via ISH analysis did not express *foxd1* or *vax2*. For these preparations, embryos were embedded dorsally on their heads and *z*-sections were acquired in an anteroposterior plane (future nasal-temporal plane) and *z*-stacks through the central retina were used to manually track cells over time traveling around the distal rim of the optic cup. Of note, like-cells (RPE or temporal neural retina) kept their nearest neighbor relationships over the period of optic cup morphogenesis. Figure 7 shows an example of a *sema6d ex6^+/+^ Tg(rx3:GFP)* eye vesicle undergoing morphogenesis, where cells at the ventral-most point of the early eye vesicle can clearly be seen to migrate around the rim of the optic cup (Fig. 7*A*,*C*, pink asterisk follows one cell). Cells in mutant eyes began to move toward the distal rim of the optic cup (*t* = 120’ for example), but by 19- to 20-hpf GFP+ (*rx3+*) neural progenitors (based on location in the ventral inner eye vesicle) stalled and showed little lateral progress. The cells could be seen to bulge out of the ventral (future temporal) inner leaflet of the eye vesicle (Fig. 7*B*,*D*, follow blue asterisk that labels one cell), and “clump” together (Fig. 7*D*).

To quantitate these data, we measured the average speed of movement of the cells in the inner leaflet over the period of optic cup morphogenesis in individual *sema6d ex6^+/+^* and *sema6d* mutant eyes. All embryos imaged in this study were genotyped postimaging. We first assessed the movement of presumptive neural progenitors (four to seven cells per embryo) of the ventral (future temporal) inner leaflet in the *Tg(rx3:GFP)* background. Presumptive neural progenitors in 17-hpf *sema6d* mutant eyes moved at ∼62% of the speed observed in controls [*sema6d ex6^+/+^*, 21.8 ± 1.4 μm/h (SEM), *n *= 4 embryos; *sema6d^ca302^*13.7 ± 1.6 μm/h, *n *= 5 embryos, *p*^k^ = 0.032, unpaired, Mann–Whitney *U* test, df = 7; Fig. 7*E*]. We then asked whether similar effects were observed with the movements of RPE progenitors (two to five cells per embryo) by assaying their behavior in a *Tg(tfec:EGFP)* background. Note that only two homozygotes were identified, thus we focused our analysis on RPE progenitors in *sema6d* heterozygous eyes (*n *= 7 embryos; Fig. 7*F*), and include the data we obtained by the tracking of cells in the two homozygous eyes for comparison purposes. We found initially at 16 hpf (14 ss) that RPE cells in both heterozygous [22.0 ± 3.4 (SEM) μm/h, *n* = 5 embryos] and homozygous (27.4 ± 3 μm/h, *n* = 2) eyes moved toward the distal rim at speeds comparable to those of *sema6d ex6^+/+^* cells (27.3 ± 1.7 μm/h, *n *= 7 embryos; Fig. 7*F*). The RPE progenitors in the heterozygote eyes slowed significantly to around half that of wild-type as optic cup morphogenesis proceeded (18 hpf, *sema6d ex6^+/+^* 22.4 ± 2.5 μm/h, *n* = 8 eyes; *sema6d^+/ca302^* 12.7 ± 1.7 μm/h, *n* = 7 embryos; *p*^l^ = 0.029, Mann–Whitney *U* test, df = 13). A similar slowing was observed in the homozygous mutant (Fig. 7*F*). These data argue that in *sema6d* mutants, cells of the temporal inner leaflet of the eye vesicle initially move toward the rim of the optic cup and then slow dramatically and fail to move around the distal rim.

### Plxna1 is a candidate receptor for Sema6d signaling during optic cup morphogenesis

**Figure 8.**
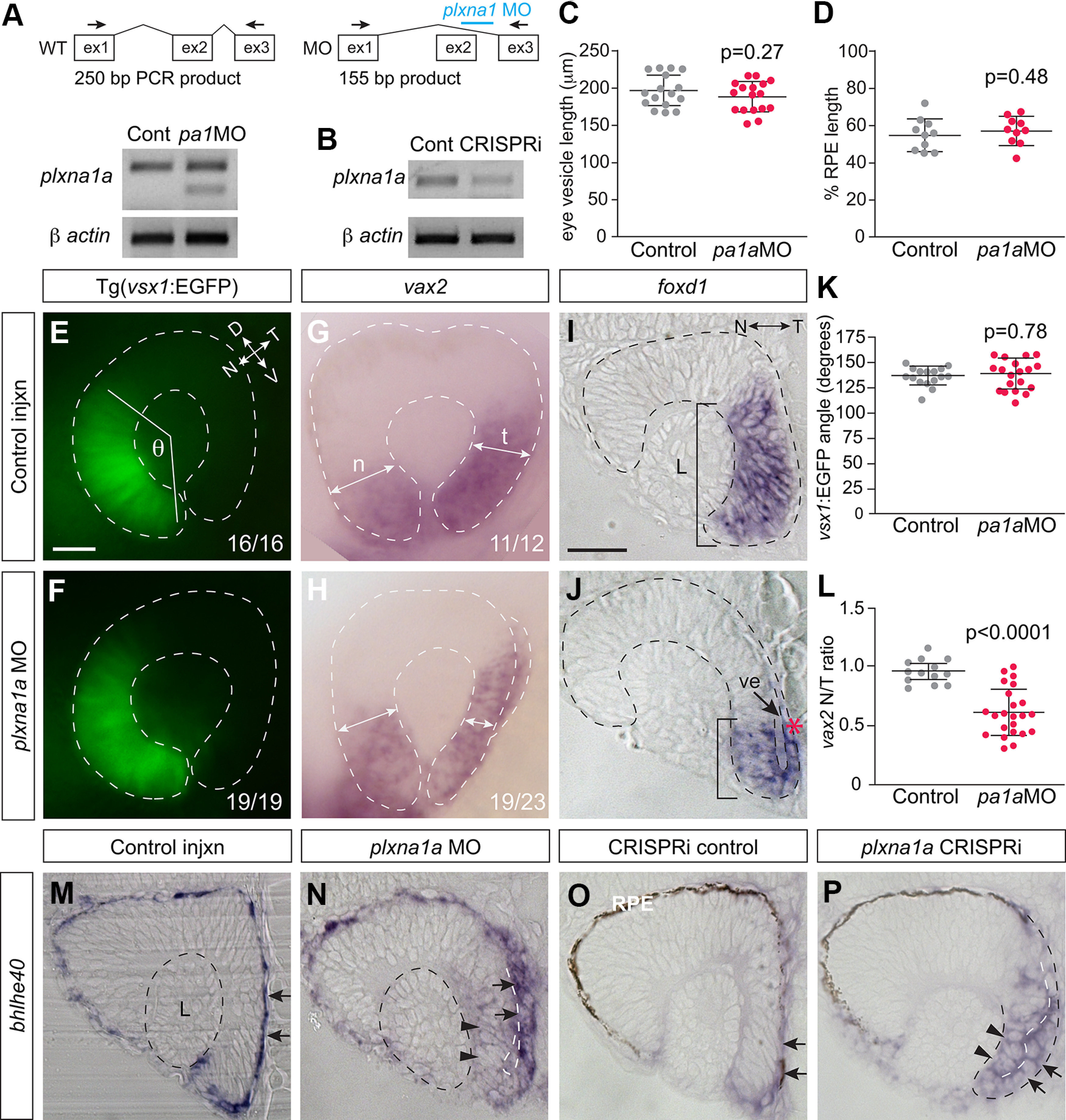
Plxna1a loss-of-function recapitulates the temporal eye defects observed in *sema6d* mutants. ***A***, ***B***, RT-PCR confirming morpholino mis-splicing (outlined in schematic) of *plxna1a* transcript (***A***), and knock down of *plxna1a* transcript in CRISPRi-injected embryos (***B***). β-Actin mRNA as loading control. ***C***, ***D***, Normal expansion of the *bhlhe40*-expressing RPE progenitor domain at the 14 ss. Mean anteroposterior eye vesicle length (***C***) and percent RPE expansion (***D***; RPE *bhlhe40+* domain length/anteroposterior eye vesicle length) are not significantly different in *plxna1a* morphants as compared with controls [*N *=* *2, control *n *=* *17, *plxna1* MO *n *=* *17; *p* values are unpaired *t* tests, df = 33 (***C***), df = 18 (***D***), error bars are SD]. ***E***, ***F***, *Tg(vsx1:GFP)* expression in the nasal retina. ***G***, ***H***, *vax2* mRNA in lateral views of a control (***G***) and a *plxna1a* morphant (***H***) 24-hpf eye. ***I***, ***J***, Transverse sections of whole-mount *foxd1+* RNA ISH of 24-hpf eyes. Early ventral (future temporal) *foxd1+* tissue undergoes rim movement into the outer leaflet in control (***I***), but in a *plxna1a* morphant remains partially in the inner leaflet (***J***; red asterisk and compare bars). Also evident is an open ventricle (arrow in ***J***) in the morphant. ***K***, The average angle formed by the lateral edges of the *vsx1* (***J***) domain to the center of the lens (θ) is similar between controls and *plxna1a* morphants (*N *=* *2; control *n *=* *16, *plxna1a* MO *n *=* *19, *p* value is unpaired *t* test, df = 33). ***L***, Ratio of the width of the temporal to nasal (t and n in ***G***) *vax2* whole-mount RNA ISH domain measured in images of lateral eyes (unpaired *t* test, *p* < 0.0001, control *n *=* *13, *plxna1a* MO *n *=* *23, error bars are SD, df = 34). ***M–P***, Transverse eye sections of whole-mount RNA ISH for *bhlhe40*+ performed on 24-hpf control embryos (***M***, ***O***) or embryos injected at the one-cell stage with either an antisense *plxna1a* morpholino (***N***) or a sgRNA against exon5 of *plxna1a* along with *dead-cas9* mRNA (CRISPRi; ***P***). RPE *bhlhe40+* progenitors elongate in control embryos (arrows in ***M***, ***O***) to line the back of the eye, and abut the lens, while the *bhlhe40* signal is expressed ectopically in the morphant and CRISPRi-injected embryos (arrowheads ***N***, ***P***), and RPE cells retain a cuboidal shape (arrows ***N***, ***P***). Embryos in ***I***, ***J***, ***M***, ***N*** were treated with 1-phenyl 2-thiourea to inhibit pigmentation of the RPE. Scale bars: 75 μm (***E***–***H***) and 50 μm (***I***, ***J***, ***M–P***). A: anterior, D: dorsal, L: lens, N: nasal, P: posterior, T: temporal, V: ventral, ve: ventricle.

Given the expression of *plxna1a* in RPE progenitors in the domain adjacent to early ventral eye vesicle neural progenitors, we asked whether Plxna1 serves as a receptor for Sema6d in optic cup morphogenesis. To do so, we perturbed Plxna1 function by injecting an antisense MO against the exon2-intron2 splice site of *plxna1a* (NCBI reference sequence XM_017353686.2) into wild-type TL embryos at the one cell stage (Fig. 8*A*). To verify the efficacy of the morpholino, we collected mRNA at 24 hpf and performed RT-PCR to confirm mis-splicing of *plxna1a* transcript (Fig. 8*A*). Perturbing Plxna1 signaling did not disrupt eye vesicle formation or elongation (Fig. 8*C*), RPE morphogenesis (Fig. 8*D*), or patterning of the early eye vesicle (data not shown). Further, the nasal retina was appropriately patterned at 24 hpf, as assessed by *vsx1* (Fig. 8*E*,*F*,*K*) and *foxg1a* (data not shown) expression. However, at 24 hpf, there was a strong phenocopy of the temporal retina defects observed with Sema6d loss-of-function: *vax2+* (*N *=* *2, 1/12 control embryos, 19/23 morphants; Fig. 8*G*,*H*) and *foxd1+* (*N *=* *2, 0/15 control embryos, 12/16 morphants; Fig. 8*I*,*J*) tissue failed to move completely around the distal rim of the temporal optic cup in the majority of morphants as compared with controls, and remained partially in the inner leaflet (Fig. 8*J*, red asterisk). We quantified this phenotype by assessing the ratio of the width of the temporal to nasal *vax2* domain, and found a significant decrease in this ratio in the *plxna1a* morphant embryos (*N *= 2; control 0.95 ± 0.1, *n *=* *13; *plxna1MO* 0.61 ± 0.2, *n *= 23; unpaired *t* test, *p*^m^ < 0.0001, df = 34; Fig. 8*L*). Further, as assessed in whole-mount (data not shown), ectopic *bhlhe40* expression within progenitors of the distal portion of the temporal neural retina was observed in 18% (*N *=* *2, *n *=* *17) of *plxna1a* morphants, as compared with 0% in control injections (*N *=* *2, *n *=* *11). The *bhlhe40* labeling phenotype was more readily assessed in transverse section, where RPE cells in the temporal region of morphant eyes were cuboidal and did not elongate properly (compare Fig. 8*M*,*N*, arrows), and ectopic *bhlhe40* label was present in the temporal neural retina (Fig. 8*N*, arrowheads). A second method of *plxna1a* mRNA knock-down via CRISPRi (Gilbert et al., 2013; Larson et al., 2013; Qi et al., 2013) corroborated these findings. A sgRNA targeting exon-5 of *plxna1a* was co-injected with *dead-cas9* mRNA at the one cell stage. mRNA was collected from control and CRISPRi-injected embryos at 24 hpf, and RT-PCR verified partial knock down of *plxna1a* mRNA transcript levels in the latter (Fig. 8*B*). We observed temporal eye defects at 24 hpf in a portion of the *plxna1a* CRISPRi-injected embryos. The ratio of the temporal to nasal *vax2* expression domain width was significantly smaller in CRISPRi embryos than control (*N *= 1; control 0.98 ± 0.05, *n *=* *5; CRISPRi 0.72 ± 0.2, *n *=* *7; unpaired *t* test, *p*^n^ = 0.012, df = 10), indicative of defects in morphogenesis of the temporal (early ventral) eye. In support, in CRISPRi-injected embryos (19%, *N *=* *2, *n *=* *21) *bhlhe40*+ RPE progenitors in the inner leaflet were cuboidal in shape (compare Fig. 8*O*,*P*, arrows) and aberrant expression of *bhlhe40* was present in the distal temporal neural retina (Fig. 8*P*, arrowheads), phenotypes not observed in any control-injected embryo (*N *=* *2, *n *=* *15). The fact that perturbing Plxna1a function produces similar eye defects as those observed with Sema6d loss-of-function is supportive of Plxna1a acting as a receptor for Sema6d signaling during optic cup morphogenesis.

### Optic cup morphogenesis may depend on Sema6d reverse signaling

**Figure 9.**
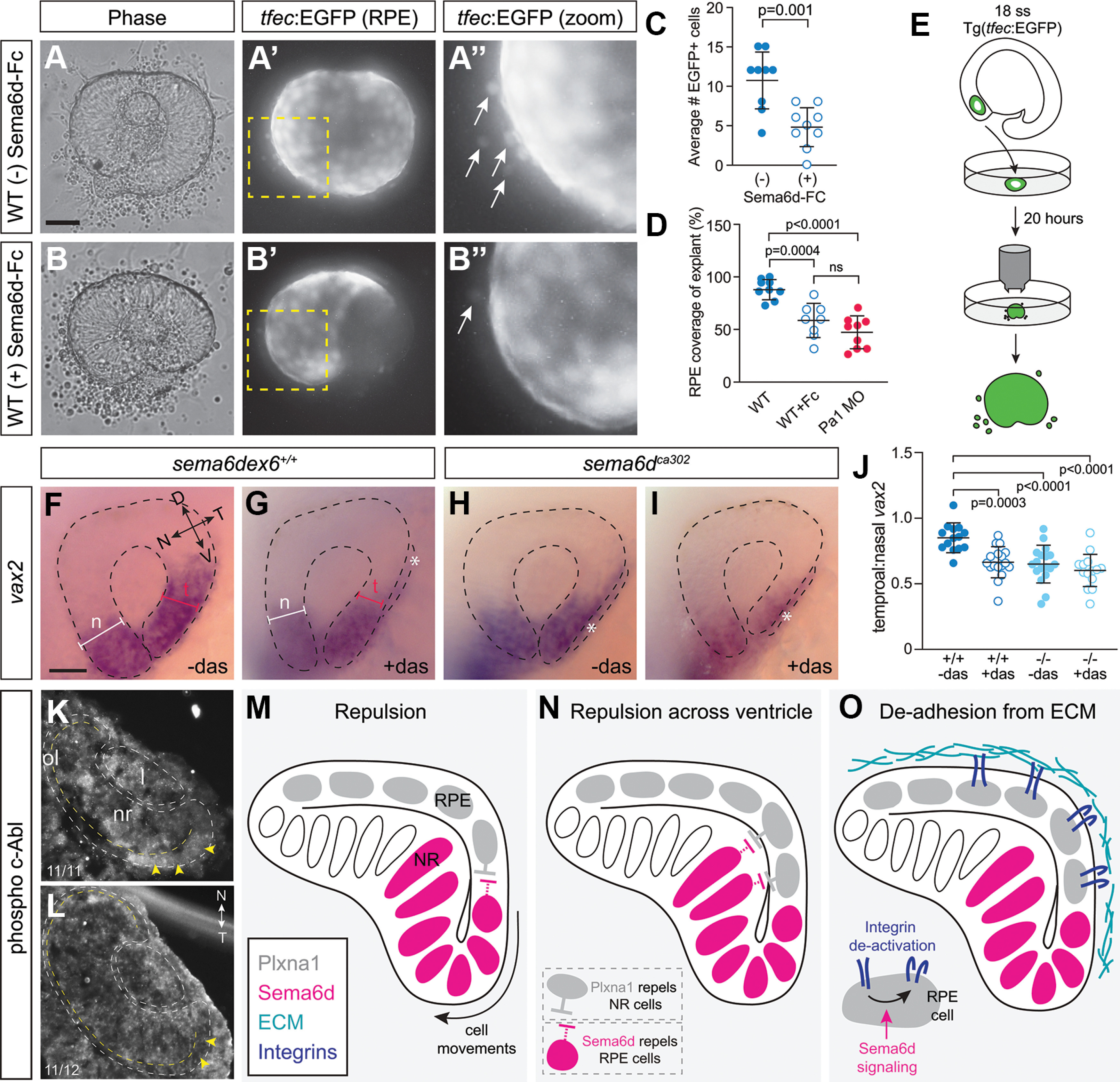
Possible Sema6d-Plxna1 repellent interactions during optic cup morphogenesis. ***A***, ***B***, EGFP+ eye vesicles from 18-ss wild-type *Tg(tfec:EGFP)* embryos were explanted and cultured in control media (***A–A’’***) or in the presence of a soluble Sema6d protein (Sema6d-Fc; ***B–B’’***). ***A’’***, ***B’’*** are magnified views of the boxed areas in ***A’***, ***B’***. ***C***, Quantitation of the average number of EGFP+ RPE cells that left the explant in the presence or absence of Sema6d-Fc (*N *=* *3; *n *=* 8–9* explants/condition each from a separate embryo, error bars are SD; unpaired *t* test, df = 16). ***D***, Percent of GFP+ RPE coverage over the explanted *Tg(tfec:EGFP)* optic cup. Eye explants cultured *in vitro* develop RPE that covers the extent of the explant, whereas those cultured in the presence of soluble Semd6d fragment do not (*N *=* *3; *n = *8–9 explants/condition, each from a separate embryo, error bars are SD; one-way ANOVA, Dunnett’s multiple comparisons test, df = 24). ***E***, Schematic of eye explant culture experiments. ***F–I***, Lateral views of *vax2* whole-mount ISH at 24 hpf. Losing Sema6d or inhibition of c-Abl with dasatinib disrupts temporal eye morphogenesis (asterisks). ***J***, Quantitation of optic cup morphogenesis defects by representing the ratio of the width of the temporal versus nasal eye (*N *=* *3; *n = *14–18 embryos/condition, error bars are SD; one-way ANOVA, Dunnett’s multiple comparisons test, df = 59). ***K***, ***L***, Immunolabeling of cryostat sections through the eye vesicle of 18-hpf wild-type (***K***) and *sema6d* mutant (***L***) embryos for the phosphorylated form of c-Abl (*N* = 2 independent experiments). Dotted yellow lines indicate the separation between the neural retina and the RPE. Yellow arrowheads point to labeling of temporal eye vesicle in wild-type, with this label largely absent in mutants. ***M***, Simple repulsion model. Our data support the possibility that Plxna1a from RPE cells activates Sema6d reverse signaling in neural retinal progenitors to promote movement of the cells of the inner eye vesicle around the distal rim of the optic cup. The possibility that Sema6d forward signals to Plxna1a-expressing RPE cells appears less likely. ***N***, Repulsion across the ventricle model. Progenitor RPE cells interact with neural retina cells to allow leaflets to slide over each other during optic cup morphogenesis. Our data support the idea that Sema6d reverse signaling is the main mode involved. ***O***, De-adhesion from ECM model. Progenitor RPE cells interact with neural retina cells to prevent over-adhesion to the ECM. Scale bars: 50 μm (***A***, ***F***).

Sema6d can signal bi-directionally, acting as either a ligand for Plxna1 or by transducing a Plxna1 signal. To determine the mode by which Plxna1 and Sema6d expressing cells communicate, we first asked whether forward signaling was possible by determining whether Sema6d is repulsive for Plxna1-expressing RPE progenitors. We explanted eye vesicles from *Tg(tfec:EGFP) sema6d ex6^+/+^* embryos (one/embryo), at a time when RPE cells express *plxna1a* mRNA (16–18 ss), and grew them in culture for 20 h in the presence or absence of soluble recombinant mouse Sema6d (Sema6d-Fc) that should be able to activate forward signaling by the Plxna1 receptor in RPE cells (Fig. 9*E*), as is suggested for chick endocardial cells (Toyofuku et al., 2004b). Cultured eye vesicle explants generally form optic cups and undergo similar morphogenesis as *in vivo* (Fig. 9*A*,*B*). The *Tg(tfec:EGFP)* label was used to identify RPE progenitors that leave the eye explant when cultured (Fig. 9*A’’*,*B’’*, arrows). Significantly fewer EGFP+ RPE cells exited the eye onto the Fibronectin substrate in the presence of Sema6d-Fc [*N *= 3; Sema6d-Fc(–) 10.8 ± 3.7 cells, *n *=* *9 explants; Sema6d-Fc(+) 4.8 ± 2.6 cells, *n *=* *9 explants; unpaired *t* test, *p*^o^ = 0.001, df = 16; Fig. 9*C*]. These data suggest that similar to what happens for a truncated soluble form of the Sema6d receptor in inhibiting the migration of chick endocardial cells (Toyofuku et al., 2004b), Sema6d-Fc can inhibit the migration of RPE progenitors.

While these data support the idea that Sema6d can forward signal through Plxna1, they do not address by which signaling mode Sem6d participates in optic cup morphogenesis. Thus, we tested the importance of Sema6d reverse signaling in temporal retinal progenitors for optic cup morphogenesis. We rejected taking a molecular approach using a dominant negative Plxna1 receptor because of difficulties in targeting expression of the mutant protein selectively to the RPE so that it would not impact other aspects of early eye development (Ebert et al., 2014). Instead, we took a pharmacological approach, where the timing of the inhibition of Sema6d signaling could be controlled tightly so as to occur just before optic cup morphogenesis. The kinase c-Abl is the only known effector of Sema6d reverse signaling, working downstream of Sema6d in macrophage polarization and embryonic chick myocardial cells (Toyofuku et al., 2004b; Kang et al., 2018). Immunolabeling for the phosphorylated activated form of c-Abl appeared downregulated in the temporal retina of 18- to 20-hpf *sema6d* mutants (Fig. 9*K*,*L*), consistent with the idea that c-Abl works downstream of Sema6d reverse signaling in temporal neural progenitors. To test this idea further, we asked whether inhibition of c-Abl phenocopied the loss of function of Sema6d. Wild-type embryos at the 16-ss stage were grown with or without 100 μm dasatinib, a pharmacological inhibitor of c-Abl (Lombardo et al., 2004), for 7 h until 24 hpf, and then processed by whole-mount ISH for the ventral/temporal domain marker *vax2* (Fig. 9*F*,*G*). In a significant number of dasatinib-treated embryos there were defects in temporal optic cup morphogenesis (Fig. 9*G*). We quantitated this effect by comparing the ratio of the width of the temporal versus the nasal retina as measured from lateral images of the *vax2* ISH processed embryos (Fig. 9*J*). Dasatinib-treated embryos exhibited a significantly smaller ratio than wild-type controls (*N *=* *3; wild-type 0.85 ± 0.11, *n *=* *14 embryos; wild-type+dasatinib 0.67 ± 0.12, *n *=* *17, *p*^p^ = 0.0003; one-way ANOVA, Dunnett’s multiple comparisons test, df = 59) because of the failure of the temporal retinal progenitors to move completely around the distal rim of the optic cup, resulting in a smaller portion of the *vax2* domain in the temporal outer leaflet (Fig. 9*G*). This phenotype is what is observed in *sema6d* mutants (*sema6d^ca302^*, 0.65 ± 0.14, *n *=* *18, *p*^q^ < 0.0001, one-way ANOVA, Dunnett’s multiple comparisons test, df = 59; Fig. 9*H*). These data support the idea that c-Abl works downstream of Sema6d in temporal retinal progenitors to promote eye morphogenesis. To further test this possibility, we performed an epistasis experiment, and treated 16 ss *sema6d^ca302^* embryos with dasatinib, with the idea being that if c-Abl works downstream of Sema6d, then c-Abl inhibition should have no additional impact on optic cup morphogenesis than the loss of Sema6d signaling itself. Indeed, this is what we observed, with a similar ratio of temporal to nasal eye width observed in *sema6d^ca302^* embryos treated with and without dasatinib (*sema6d^ca302^*, 0.65 ± 0.14, *n *=* *18; *sema6d^ca302^*+dasatinib, 0.60 ± 0.13, *n *=* *14; df = 59; Fig. 9*H–J*).

While c-Abl is the only kinase reported to date to work downstream of Sema6d (Toyofuku et al., 2004b; Kang et al., 2018), dasatinib can inhibit other kinases and so we were unable to rule out the possibility that dasatinib impacts forward signaling through a kinase active downstream of Plxna1 (Lombardo et al., 2004). To further explore the participation of Sema6d reverse signaling, we revisited the Sema6d-Fc treatment of wild-type eye explant cultures. In the developing chick heart, Sema6d mediates both forward and reverse signaling (Toyofuku et al., 2004b). In this system, misexpression of soluble Sema6d in the embryonic heart is reported to both promote forward signaling and block reverse signaling (Toyofuku et al., 2004a,b). As such, we expected that Sema6d-Fc should bind the Plxna1a of RPE cells and promote forward signaling via activation of the receptor, but also block reverse signaling, in that the Sema6d-Fc occupied RPE-expressed Plxna1a receptor would no longer be available to interact with Sema6d in temporal neural progenitors. Note that while we assayed above the behavior of RPE cells alone (Fig. 9*C*), here, we assayed optic cup morphogenesis, which depends on the movements of temporal neural retinal progenitors. We reasoned that if optic cup morphogenesis is controlled by Sema6d activating Plxna1 forward signaling, then Plxna1a signaling would be activated by both temporal progenitor Sema6d and the soluble Sema6d-Fc and optic cup morphogenesis would occur normally. If instead reverse signaling is key, by binding to the Plxna1a of RPE progenitors the soluble Sema6d-Fc would act as an antagonist and block the ability of Plxna1a to interact with and activate endogenous Sema6d reverse signaling. As such, optic cup morphogenesis would be disrupted. As described above, explanted eyes from *Tg(tfec:EGFP)* wild-type 16–18 ss fish were treated with and without soluble Sema6d-Fc. Eye buds from *Tg(tfec:EGFP)* embryos injected at the one cell stage with the *plxna1a* morpholino were used as a positive control, as we expected optic cup morphogenesis defects with impaired Plxna1a signaling. Wild-type explants underwent optic cup morphogenesis to produce an optic cup surrounding the lens, and with GFP+ RPE cells that expanded and covered almost the entire optic cup, as they would have *in vivo* (Fig. 9*A****’***). The degree of optic cup covered by the GFP+ RPE was measured, to represent the extent to which optic cup morphogenesis proceeded to completion (Fig. 9*D*). This measure was reduced significantly in the *plxna1a* morpholino-treated explants, agreeing with the *in vivo* data indicating that Plxna1a function is required for normal optic cup morphogenesis (*N *=* *3; wild type, 88.4 ± 9.1%, *n *=* *9 explants; *plxna1a* MO 47.0 ± 15.4%, *n *=* *9; *p*^r^ < 0.0001, one-way ANOVA, Dunnett’s multiple comparisons test, df = 23). Treatment of wild-type explants with Sema6d-Fc produced a similar eye morphogenesis defect, notably with the RPE failing to cover the temporal optic cup (58.6 ± 16%, *n *=* *8 explants, *p*^s^ = 0.0004; Fig. 9*B’*,*D*). These data, alongside the c-Abl downregulation in temporal progenitors of *sema6d* mutants, support the idea that Sema6d reverse signaling promotes optic cup morphogenesis.

## Discussion

Little is known about the cellular and molecular interactions that drive optic cup morphogenesis. We reveal an important role for Sema6d-mediated communication between retinal and RPE progenitors in the flow of the inner leaflet of the eye vesicle around the distal rim of the invaginating optic cup. Specifically, we find *sema6d* mRNA in ventral neural retina progenitors (future temporal) of the inner leaflet, while the adjacent RPE progenitors of the dorsal inner leaflet express Plxna1a mRNA. Loss of Sema6d signaling, either by CRISPR/Cas9 mutagenesis of *sema6d*, transient Plxna1a knock-down, or c-Abl inhibition, impairs the movements of both ventral retinal cells and their RPE neighbors. Our study identifies for the first time that interaction of RPE and neural retina progenitors, via a Sema6d-dependent mechanism, is a key modulator of optic cup morphogenesis.

During optic cup morphogenesis, *sema6d+* neural progenitors of the ventral inner vesicle migrate around the distal rim of the vesicle to form the mature temporal neural retina, and *plxna1a+* RPE cells elongate and come to comprise the entire inner leaflet of the optic cup. When Sema6d signaling is impaired, these processes are disrupted. Ventral *foxd1/vax2*+ progenitors accumulate in a disorganized fashion at the lateral edge, and significant numbers of these progenitors fail to move normally into the retina proper. In an associated manner, RPE cells of the inner leaflet do not elongate and stretch to the temporal edge of the lens. Time-lapse imaging reveals that morphogenesis initiates properly, with neural progenitors moving toward the rim and RPE cells elongating. Rim movement, however, proceeds more slowly and then stalls as cells of the ventral inner leaflet clump and form a disorganized bulge.

The morphogenesis defects appear specific to Sema6d and potentially involve a known Sema6d partner, Plxna1a (Toyofuku et al., 2004a,b). First, two distinct mutant alleles exhibit the same phenotype, which is restricted to the eye domains that express *sema6d* and *plxna1a*: defects are seen in RPE expansion and ventral eye vesicle morphogenesis, but not morphogenesis of dorsal (future nasal) eye. Second, knock down of Plxna1a, via two distinct means, produces temporal eye phenotypes similar to those of *sema6d* mutants.

Cellular interactions with Integrin of the basal lamina drive temporal progenitor migration during optic cup morphogenesis (Sidhaye and Norden, 2017). Our data suggest that Sema6d provides an additional mechanism during morphogenesis, and point to Plxna1a in the RPE as the interacting receptor. The fact that loss of Sema6d or Plxna1a alone produce similar morphogenesis defects, argues that RPE and retinal progenitors have to interact for normal morphogenesis. Thus, we propose that RPE expansion does not depend entirely on the cells being stretched passively by the rim movement of retinal progenitors. Instead, RPE and neural progenitors must signal to each other, through a cell-cell contact-mediated process. Of note, the contraction of RPE cells may also contribute to morphogenesis by pushing the neural progenitors around the rim.

Sema6d, and other transmembrane Sema6s, mediate forward signaling through a Plxn, and reverse signaling through their intracellular domain (for review, see Casazza et al., 2007). We expect that no reverse signaling occurs in mutants, even if protein is made, in that mutant Sema6d is predicted to lack transmembrane and intracellular domains. Forward signaling is likely also impacted in the mutants, because even if truncated Sema6d is present it would lack the intact Sema domain for binding Plxna (Janssen et al., 2010; Nogi et al., 2010). Forward signaling could normally be active in RPE progenitors. Indeed, a parsimonious explanation for our culture data is that Sema6d-Fc inhibits RPE migration via activation of Plxna1 forward signaling, although alternative explanations are possible. Yet, our data speak to an important role for Sema6d reverse signaling in optic cup morphogenesis. Soluble Sema6d was shown to block events in the embryonic chick heart that depend on Sema6d reverse signaling, while promoting those that require forward signaling (Toyofuku et al., 2004a,b). Based on these observations, our Sema6d-Fc data, where optic cup morphogenesis is inhibited, argue that morphogenesis depends on Sema6d reverse signaling. In agreement, the activation of a known Sema6d reverse signaling downstream mediator, c-Abl, appears downregulated in mutant temporal retinal progenitors, and a pharmacological inhibitor of this kinase produces a morphogenesis defect similar to that of *sema6d* mutants. Thus, our data support a role for reverse signaling in eye morphogenesis but do not exclude the involvement of forward signaling.

One could imagine a simple mechanism by which Plxna1a+ RPE cells reverse signal to Sema6d-expressing neural progenitors and help push a front of ventral neural tissue around the distal rim (Fig. 9*M*). An alternate possibility is a “tissue sliding” model (Fig. 9*N*). Cells in the inner and outer leaflets slide past each other in opposite directions during optic cup morphogenesis; RPE progenitors move laterally, while neural progenitors travel medially on entry into the retina (Kwan et al., 2012; Heermann et al., 2015). A sliding of the anterior mesoderm and overlying ectoderm occurs during *Xenopus* gastrulation (for review, see Hammerschmidt and Wedlich, 2008), with Eph-ephrin repulsion preventing the two tissues from adhering tightly (Park et al., 2011). Similarly, Sema6d-Plxna1a repulsion could allow the inner and outer eye vesicle leaflets to move past one another, with repulsion preventing cells in opposing leaflets from sticking to each other too tightly. Time-lapse imaging indicates that neural and RPE progenitors are in close proximity and likely physically interact, as the basal lamina lines the opposite side of the two epithelia (Sidhaye and Norden, 2017).

A “de-adhesion” mechanism described in *Drosophila* proposes an alternate means to reduce RPE cell “stickiness,” where transmembrane Sema-1a binds to PlxnA and blocks Integrin-extracellular matrix (ECM) interactions (for review, see Yang and Terman, 2013). While, Sema6d activation of RPE Plxna1a might similarly prevent tight adherence of the RPE to Laminin (Fig. 9*O*; Sidhaye and Norden, 2017), this model seems less likely in that our data speak against a significant role for Sema6d forward signaling. Regardless of the exact mechanism, we propose a model whereby Sema6d promotes the efficient movement of cells via reducing adhesive contacts, either between cells or with the ECM.

Interestingly, our data suggest that the neural retinal cells of the inner leaflet normally transiently express RPE markers. Indeed, at 22 hpf, these cells are positive for both *foxd1* and EGFP in a *Tg(tfec:EGFP)* background. *tfec/bhlhe40/*EGFP are more weakly expressed by these temporal progenitors than by RPE cells, and expression is quickly lost once the progenitors settle within the temporal retina. The continued expression of RPE markers by temporal progenitors in *sema6d* mutants, both for progenitors that complete their movement into the retina and for those still stuck in the inner leaflet, could reflect a role of Sema6d signaling in cell fate specification. If true, a defect in the movement of temporal progenitors could occur secondary to a failure to acquire the appropriate identity. While possible, our data and the literature support instead that dys-regulated RPE gene expression occurs secondary to the mislocalization of temporal cells. First, Sema6s are known as regulators of cell movements (for review, see Battistini and Tamagnone, 2016; Alto and Terman, 2018), with no reports to our knowledge of direct roles in cell fate specification. Second, wild-type temporal progenitors also exhibit a mixed expression of RPE and neural markers, but lose this expression once they settle in the retina. Thus, marker co-expression is a feature of normal eye development, and not unique to the mutant background. Instead, we propose that on arrival into the retina, temporal retinal progenitors either receive an extracellular signal to inhibit RPE gene expression, or lose a signal that maintains RPE marker expression. The ectopically located temporal progenitors in the *sema6d* mutant would fail to receive these positional signals.

Other Sema6s are expressed in the developing eye vesicle. Indeed, Sema6a/Plxna2 are required for vesicle extension (Ebert et al., 2014). Thus, distinct Sema6s may coordinate the different cell movements that drive eye morphogenesis. While multiple *Sema6* genes are expressed in the postnatal mouse retina (Matsuoka et al., 2013), only loss of SEMA6A affects retinal circuits (Sun et al., 2013). No morphogenesis phenotype has been reported in *Sema6* mouse mutants. Possibly, SEMA6s act redundantly to each, or other repellent signaling pathways function in their place. Alternatively, roles in eye morphogenesis may have been overlooked, given that the loss of Sema6d in zebrafish does not produce a change in eye size.

The cellular movements involved in eye morphogenesis are characterized (Picker et al., 2009; Kwan et al., 2012; Heermann et al., 2015; Cechmanek and McFarlane, 2017; Sidhaye and Norden, 2017), and we know that acto-myosin contractility and cellular interactions with the ECM are required (Nicolás-Pérez et al., 2016; Sidhaye and Norden, 2017). Here, we provide data to support a need for communication between RPE and retinal progenitors via Sema6d/Plxna1a, and implicating cell-cell contact mediated signaling in optic cup morphogenesis. The expression of *SEMA6s* in other embryonic tissues (Xu et al., 2000; Toyofuku et al., 2004a; Koestner et al., 2008; Shalom et al., 2019) suggests they may play similar roles in mediating cell-cell and/or cell-ECM interactions that underlie morphogenesis of other embryonic tissues.

## Synthesis

Reviewing Editor: Lynda Erskine, University of Aberdeen Decisions are customarily a result of the Reviewing Editor and the peer reviewers coming together and discussing their recommendations until a consensus is reached. When revisions are invited, a fact-based synthesis statement explaining their decision and outlining what is needed to prepare a revision will be listed below. Note: If this manuscript was transferred from JNeurosci and a decision was made to accept the manuscript without peer review, a brief statement to this effect will instead be what is listed below.

Thank you for submitting your manuscript to eNeuro and providing information on the changes made in response to the previous reviews and reviewing editor’s comments. My decision is based on both your rebuttal of the previous reviews and reading of the revised manuscript.

Careful and detailed analyses of optic cup morphogenesis in Sema6d mutants and Plxna1a morphants have been performed, demonstrating that the phenotypes are similar. Overall, the findings are clearly presented and the data convincing, providing new information on the mechanisms controlling optic cup morphogenesis. The main issue that remains is the directionality of signalling. I agree with the statement in the rebuttal that “whether Sema6d acts in a reverse or forward manner is not important to our demonstration of Sema6d’s relevance to eye morphogenesis, and to the novelty of our work in identifying a molecular mechanism involving interactions between progenitors that support optic cup morphogenesis.” However, this needs to be brought out more in the Discussion and care taken not to oversell the significance of some of the findings. Additionally, the description of the experiments undertaken to try and distinguish between roles for forward and reverse signalling would benefit from some clearer explanations and considerations of alternative explanations for the findings.

Below is a list of points that I would like you to consider prior to publication in eNeuro. All of these should be able to be addressed through text changes.

1. I found the section “Sema6d reverse signalling is required for optic cup morphogenesis” a bit confusing on initial read through (line 479 onwards). This is particularly the case because Sema6d-Fc is used initially to stimulate forward signalling and then later to block reverse signalling. The logic behind being able to use the same reagent to test for both types of signalling in the same tissue needs to made clearer.

Through addition of Sema6d-Fc to cultured eye vesicles it is concluded that Sema6d can act as a repellent for RPE progenitors (ie forward signalling). Could blocking of reverse signalling also explain this phenotype? For example, through inhibiting ability of RPE cells to delaminate as opposed to a repellent effect? Or secondary to the morphogenesis defects reported later in the section? Alternative explanations such as these need to be considered and, if not likely, the rational clearly explained. As presented, I am not convinced that this experiment provides strong evidence in support of forward (or reverse) signalling.

The finding that c-Abl may work downstream of Sema6d - it would be helpful if it was stated explicitly that this finding is consistent with reverse signalling.

Lines 540 onwards - to help readers understand the rational/logic of the experiments and the interpretation of the data it would be helpful to move this explanation of the anticipated effects of Sema6d-Fc on forward versus reverse signalling to before the description of the relevant experiments.

Although Sema6d-Fc can both potentially activate forward signalling and block reverse signalling it is referred to in the Discussion as “a reagent that should block reverse signalling”. (line 593).

2. The data show potential involvement of reverse signalling but do not definitively demonstrate that forward signalling is not required. Statements in the discussions such as “our data speak against a significant role for forward signalling” (line 619) should be toned down or the rational for this conclusion made clearer. Similarly, it is possible, but not definitively demonstrated that reverse signalling “plays the predominant role” (line 600). As stated in the rebuttal “whether Sema6d acts in a reverse or forward manner is not important to our demonstration of Sema6d’s relevance to eye morphogenesis”. Focusing the Discussion more on the importance of the findings for eye development (identification of a molecular mechanism involving interactions between progenitors that support optic cup morphogenesis) rather than on whether forward or reverse signalling is more critical may help to address this.

3. Legend Figure 8 - the use of small letters alongside upper case panel labels is rather confusing (e.g. “mean antero-posterior eye vesicle length (b, F”), “(a/b, G)”. The legend should be modified to explain more clearly what ’b” and “a/b” are referring to.

4. Methods sections - Experimental Design and Statistical Analyses (line 201 onwards): The approaches that were used to confirm the data are normally distributed should be stated (t-tests and ANOVA are only appropriate for normally distributed data).

5. Line 352 - “in the occasional mutant” - the number of embryos with this rare phenotype relative to the total number analysed should be stated. Similarly, the proportion with the “more common” phenotype (line 354) should be stated.

6. Figures 3, 4, 5 - the legends should explain what the numbers in the bottom right corner of each panel indicate.

7. With regard to the point raised in the previous reviews of moving Table 1 to Extended Data, I am in favour of this table being included in the main manuscript. It contains important information on the regions recognised by each riboprobe. The information also will be important should anyone wish to generate comparable riboprobes.

## Author Response

Response to Review:

We appreciate the careful read over of the manuscript by the Senior Editor. Making sure that

we have clearly described the experiments and their rationale, and not over interpreted the

data is important. The Editor’s comments have helped considerably in that respect.

1. I found the section “Sema6d reverse signalling is required for optic cup morphogenesis” a bit

confusing on initial read through (line 479 onwards). This is particularly the case because

Sema6d-Fc is used initially to stimulate forward signalling and then later to block reverse

signalling. The logic behind being able to use the same reagent to test for both types of

signalling in the same tissue needs to made clearer.

Thank you for catching this issue. We provide more clear explanation as to why we would

expect Sema6d-Fc to both promote forward signaling and block reverse signaling. Importantly,

it has already been shown in the chick embryonic heart that a soluble form of Sema6d

promotes events that depend on Sema6d forward signaling (ventricular expansion) but blocks

those that depend on reverse signaling (trabeculation) (Toyofuku et al., 2004a; Toyofuku et al.,

2004b). We provide better use of these references in explaining the rationale of our

experiments.

Through addition of Sema6d-Fc to cultured eye vesicles it is concluded that Sema6d can act as a

repellent for RPE progenitors (ie forward signalling). Could blocking of reverse signalling also

explain this phenotype? For example, through inhibiting ability of RPE cells to delaminate as

opposed to a repellent effect? Or secondary to the morphogenesis defects reported later in the

section? Alternative explanations such as these need to be considered and, if not likely, the

rational clearly explained. As presented, I am not convinced that this experiment provides

strong evidence in support of forward (or reverse) signalling.

Because the phenotype we measured (Figure 9C) pertains to individual RPE cells (which is not

the case for optic cup morphogenesis that we assay for Figure 9D, which requires the

movements of neural retinal progenitors), the most parsimonious explanation is that Sema6dFc directly affects Plxna1 signaling in RPE cells. Invoking inhibition of reverse signaling then has

to involve a bi-directional signaling process, where RPE signals to temporal neural progenitors,

that then signal back to RPE cells to affect their behaviour. The more complicated nature of

invoking Sema6d-Fc blocking reverse signaling to explain an RPE cell phenotype is illustrated

when we work through our forward signaling suggestion, and the two possible explanations

provided by Dr. Erskine. Note that our “forward” signaling explanation is a gain-of-function

explanation, whereas the blockage of reverse signaling by Sema6d is a loss-of-function

explanation, and requires an existing biological process to be inhibited.

a) Sema6d-Fc mimics Plxna1 forward signaling by acting via Plxna1a on RPE cells. Such a

scenario was shown for chick endothelial cells (Toyofuku et al., 2004a; Toyofuku et al.,

2004b). In an in vitro setting (not in vivo) some RPE cells extend from the eye explant.

Sema6d-Fc treatment blocks the ability of RPE cells to do so. Importantly, Sema6d

forward signaling may not occur in vivo, rather this gain-of-function explanation (where 2

we provide exogenous Sema6d-Fc), provides a proof of principle that Sema6d can act as

a repellent for RPE cells.

b) Sema6d-Fc inhibits the ability of RPE cells to delaminate by blocking reverse signaling.

Here one would have to imagine a scenario where normally RPE cells use Plxna1a to

activate Sema6d reverse signaling in temporal neural retina progenitors. These cells

would then have to signal back to RPE cells and cause them to delaminate. Unlike

scenario “a” above, this would be a normal biology, and it would be the soluble Sema6dFc that would be interfering; soluble Sem6d-Fc would block Sema6d reverse signaling

and the signal back to RPE cells would be lost and RPE cells would not delaminate. We

find this explanation less attractive than the one suggested in “a” in that it requires a

considerably more complicated signaling pathway. Second, we have never seen

evidence in our time lapse videos (here and in Cechmanek et al., 2017) that normally

RPE cells delaminate from WT eyes undergoing morphogenesis.

c) The failure of GFP+RPE cells to emerge from the explant is secondary to the Sema6d-Fc

causing a morphogenesis defect. We also find this explanation less attractive than in “a"

in that it would have GFP+ RPE cells leaving a WT explant undergoing normal

morphogenesis but not doing so with an explant that had not undergone proper

morphogenesis (Sema6d-Fc treated). We saw the opposite.

We would prefer to keep the most parsimonious explanation, and one supported by the

literature (Toyofuku et al., 2004a; Toyofuku et al., 2004b), of the Sema6d-Fc effects on GFP+

RPE cells, and not involve reverse signaling. Indeed, in the cardiovascular system Sema6d is

known to inhibit the migration of endocardial cells via forward signaling (Toyofuku et al.,

2004b). Nonetheless, we have toned down the conclusion we make about Sema6d forward

signaling and repulsion, and make it clear that in the assay in Figure 9C we are assaying RPE

behaviour, while the assay in Figure 9D we assay optic cup morphogenesis, which depends on

the behaviour of neural retinal progenitors.

The finding that c-Abl may work downstream of Sema6d - it would be helpful if it was stated

explicitly that this finding is consistent with reverse signalling.

We now make this statement explicitly.

Lines 540 onwards - to help readers understand the rational/logic of the experiments and the

interpretation of the data it would be helpful to move this explanation of the anticipated

effects of Sema6d-Fc on forward versus reverse signalling to before the description of the

relevant experiments.

We have made these changes.

Although Sema6d-Fc can both potentially activate forward signalling and block reverse

signalling it is referred to in the Discussion as “a reagent that should block reverse signalling”.

(line 593).3

We have rewritten the relevant paragraph in the Discussion.

2. The data show potential involvement of reverse signalling but do not definitively

demonstrate that forward signalling is not required. Statements in the discussions such as “our

data speak against a significant role for forward signalling” (line 619) should be toned down or

the rational for this conclusion made clearer. Similarly, it is possible, but not definitively

demonstrated that reverse signalling “plays the predominant role” (line 600). As stated in the

rebuttal “whether Sema6d acts in a reverse or forward manner is not important to our

demonstration of Sema6d’s relevance to eye morphogenesis”. Focusing the Discussion more on

the importance of the findings for eye development (identification of a molecular mechanism

involving interactions between progenitors that support optic cup morphogenesis) rather than

on whether forward or reverse signalling is more critical may help to address this.

We rewrote the sections in the Results and Discussion that pertain to the Sema6d forward and

reverse signaling. We provide better explanation for the experiments, and have toned down

our conclusions.

3. Legend Figure 8 - the use of small letters alongside upper case panel labels is rather

confusing (e.g. “mean antero-posterior eye vesicle length (b, F”), “(a/b, G)”. The legend should

be modified to explain more clearly what “b” and “a/b” are referring to.

We have removed “a” and “b” from both the figure and the figure legend.

4. Methods sections - Experimental Design and Statistical Analyses (line 201 onwards): The

approaches that were used to confirm the data are normally distributed should be stated (t tests and ANOVA are only appropriate for normally distributed data).

We have made the necessary adjustments. All data passed the Shapiro-Wilk test for normality.

We used a Mann Whitney non-parametric test for analysis of the time lapse microscopy data in

that sample sizes were small and so it was not really possible to test for normality. We now

make this clear.

5. Line 352 - “in the occasional mutant” - the number of embryos with this rare phenotype

relative to the total number analysed should be stated. Similarly, the proportion with the “more

common” phenotype (line 354) should be stated.

We provide this information.

6. Figures 3, 4, 5 - the legends should explain what the numbers in the bottom right corner of

each panel indicate.

We have added this explanation to the figure legends.

7. With regard to the point raised in the previous reviews of moving Table 1 to Extended Data, I 4

am in favour of this table remaining in the main manuscript. It contains important information

on the regions recognised by each riboprobe. The information also will be important should

anyone wish to generate comparable riboprobes.

Table 1 will be included in the main manuscript.
